# Total nitrogen and phosphorus loads in surface runoff from urban land use (city of Lublin) under climate change

**DOI:** 10.1007/s11356-024-34365-9

**Published:** 2024-07-17

**Authors:** Ewa Szalińska, Elżbieta Jarosińska, Paulina Orlińska-Woźniak, Ewa Jakusik, Wiktoria Warzecha, Wioletta Ogar, Paweł Wilk

**Affiliations:** 1 AGH University of Krakow, A. Mickiewicza Av. 30, 30-059 Krakow, Poland; 2 PK Cracow University of Technology, Warszawska 24, 31-155 Krakow, Poland; 3 Institute of Meteorology and Water Management - National Research Institute, Podleśna 61, 01-673 Warsaw, Poland

**Keywords:** Urban area, Nutrient loads, Surface runoff, SWAT, Climate change

## Abstract

An expansion of impervious surfaces in urban areas leads to increases of nutrient loads discharged with the surface runoff to receivers. A study of a different density of urban development impact on total nitrogen (TN) and phosphorus (TP) loads from the city of Lublin (eastern Poland) with the use of the SWAT (Soil & Water Assessment Tool) model was performed. To distinguish between areas with high and low density of urban development (UHD and ULD), a special analysis of hydrological parameters has been proposed. Moreover, to investigate the impact of climate change, four variant scenarios were taken into account, combining the RCP (representative concentration pathway) 4.5 and 8.5 forecasts and the adopted time horizons (2026–2035 and 2046–2055). The results showed a much higher share of TN and TP from UHD compared to ULD (86%—32 022 kg/year and 89%—2574 kg/year, respectively). In addition, the variant scenarios showed that the forecasted increase in precipitation and temperature will result in increased loads of nutrients from UHD and ULD up to 30%. Furthermore, the current increase of inhabitant number, due to the Ukrainian war migration and the common tendency to convert agricultural land to residential areas, could contribute to further expansion of UHD and ULD areas and an additional increase of nutrient loads.

**Graphical abstract:**

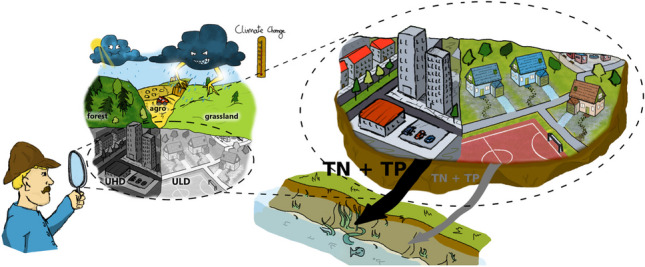

**Supplementary Information:**

The online version contains supplementary material available at 10.1007/s11356-024-34365-9.

## Introduction

The increasing development of urban areas causing growth of the share of impervious surfaces and reduction of vegetation are major factors of excessive amounts of surface runoff and its faster outflow to receivers (Fox et al. 2012; Dams et al. 2013; Jarosińska 2016; Jarosińska and Gołda 2020; Sonu et al. 2022; Kirker & Toran 2023; Nguyen et al. 2023; Oswald et al. 2023). Moreover, these processes will be intensified by climate change bringing an increasing number of weather anomalies, including intense precipitation (Larsen et al. 2009; Jarosińska 2019; Shenoy et al. 2022; Clarke et al. 2022; Sun et al. 2022) and having a significant impact on the resilience and vulnerability of urban areas (Xie et al. 2023). The intensification of urbanization alters hydrological processes taking place in urban areas, e.g. surface runoff (Xu et al. 2020), evapotranspiration, infiltration (Wang et al. 2021) and groundwater recharge (Tubau et al. 2017). Excessive urbanization can also promote a significant increase of pollutants, i.e. nitrogen and phosphorus from nonpoint sources, causing eutrophication and hypoxia in rivers (Carey et al. 2013; Garnier et al. 2013; Zheng et al. 2021; Ly et al. 2021; Li et al. 2023). Therefore, even though large urban agglomerations cover relatively small portions of catchments, their impact on adjacent riverine networks can be overwhelming and degrading (Lyu et al. 2019; Liu et al. 2021). Catchment models can be very helpful in predicting and estimating the scale of such problems, provided that the representation of urban areas in such models is accurate and takes into account their heterogeneity.

One of the commonly used catchment models to simulate the quality and quantity of surface waters and to predict the environmental impact of anthropogenic activities and climate change is the SWAT model (Wang et al. 2019). Although originally created mainly for agriculture, it also takes into account a whole range of different types of land use, including various kinds of urban development with different density. However, it should be noted that the SWAT model output files are not directly adapted to distinguish between them. In addition, representation of the research on the impact of impervious areas on the current and future surface runoff quality is still quite limited. So far, studies using the SWAT model for urban areas have been mainly limited to hydrological processes (Yang and Li 2011; Sisay et al. 2017; Li and DeLiberty 2020; Busico et al. 2020; Koltsida et al. 2021; Whitehurst et al. 2021), often treating the city as a homogeneous area. Consequently, there are still unanswered questions regarding the current and future pollutant loads (Krimsky et al. 2021; Yazdi et al. 2021) and their differentiation depending on the type of urban development. Therefore, in this study, we offer a novel methodology, based on simple hydrological parameters, allowing the distinction between urban areas with high and low density of urban development (UHD and ULD). This methodology will help to fill the knowledge gap on urban nutrient loads but also will expand the possibilities of modeling tool. However, the main stimuli to undertake the described actions were results of our previous research showing a noticeable increase of nutrient loads (especially phosphorus) in parts of the catchment occupied by urbanized areas (Orlińska-Woźniak et al. 2021) and divergent reaction of the urban areas to climate change scenarios when compared with other diffuse sources (Bojanowski et al. 2023).

The goal of this study is to quantify nutrient loads released with the surface runoff from different types of land use (UHD, ULD, agriculture, forest and grassland) in the municipality of Lublin (Bystrzyca Lubelska River catchment; eastern Poland), with a particular emphasis on the heterogeneity of urban development. Simulations using a digital platform—Macromodel DNS/SWAT (Discharge-Nutrient-Sea/Soil & Water Assessment Tool) (Wang et al. 2019)—were performed for nine subbasins with a varied share of UHD and ULD areas. Moreover, to estimate future changes in nutrient loads from these types of land use, the impact of climate change has been investigated in this study. Additionally, the impact of potential urban area expansion due to the population migration as a result of the war in Ukraine has been investigated in the context of the nutrient load increase.

## Material and methods

### Study area

**Fig. 1:**
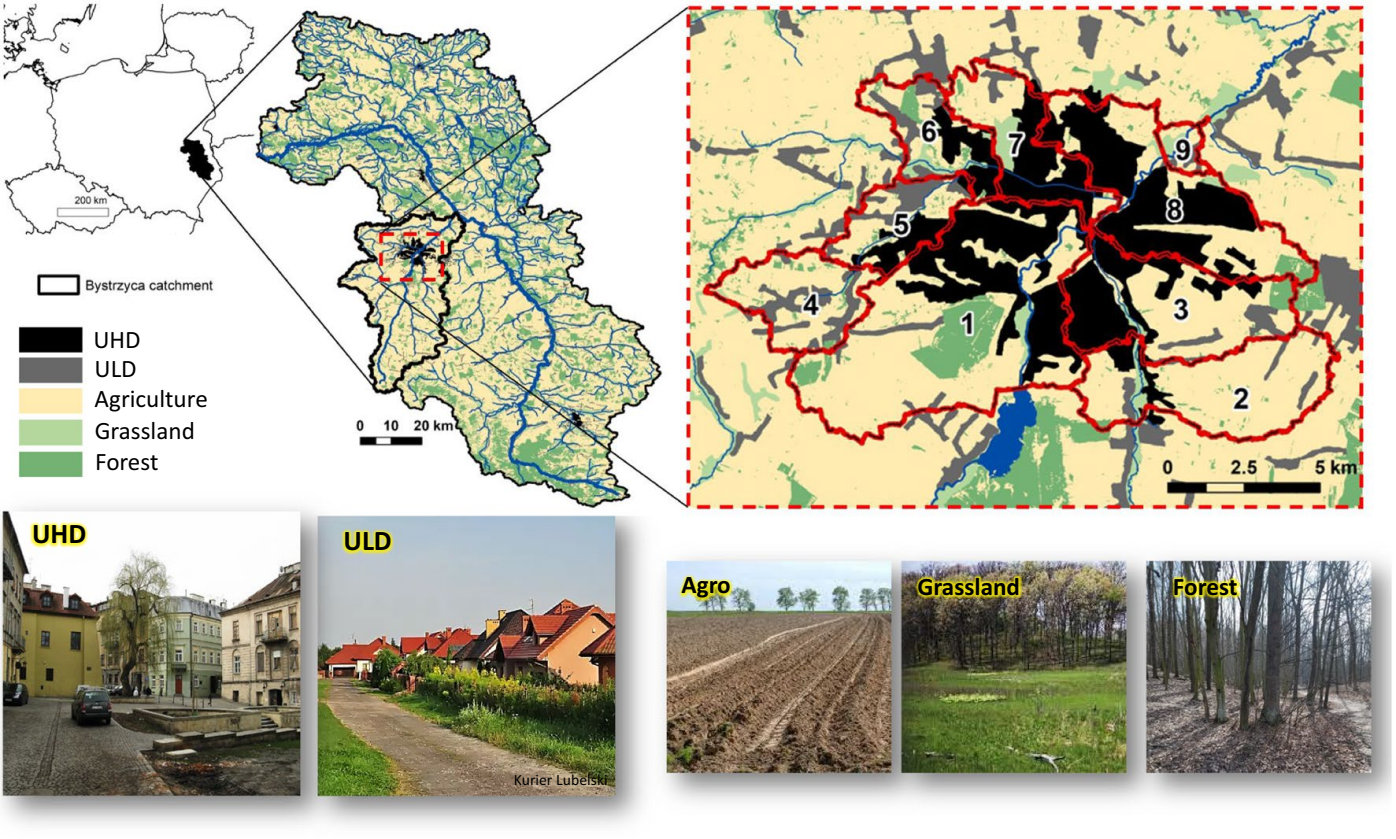
The Wieprz River basin with localization of the city of Lublin and division into subbasins

Lublin is one of the biggest Polish municipalities in the south-eastern part of the country (Zgłobicki et al. 2018). This city is located by the Bystrzyca Lubelska River, which constitutes a left-bank tributary of one of the largest rivers in Poland: Wieprz (360 km of length and over 10,000 km^2^ of catchment area) (Fig. 1). The SWAT model, originally built for the entire area of the Wieprz River basin, was used for temporal and spatial studies of the quantity and quality of surface runoff. The hydrological division offered by this tool allowed us to separate those parts of the basin that include urban parts of Lublin and the adjacent undeveloped areas (Fig. 1). Finally, nine subbasins with a total area of 15,908 ha and a population of over 260,000 were selected, which formed the research area, later named “the city of Lublin.”

**Fig. 2:**
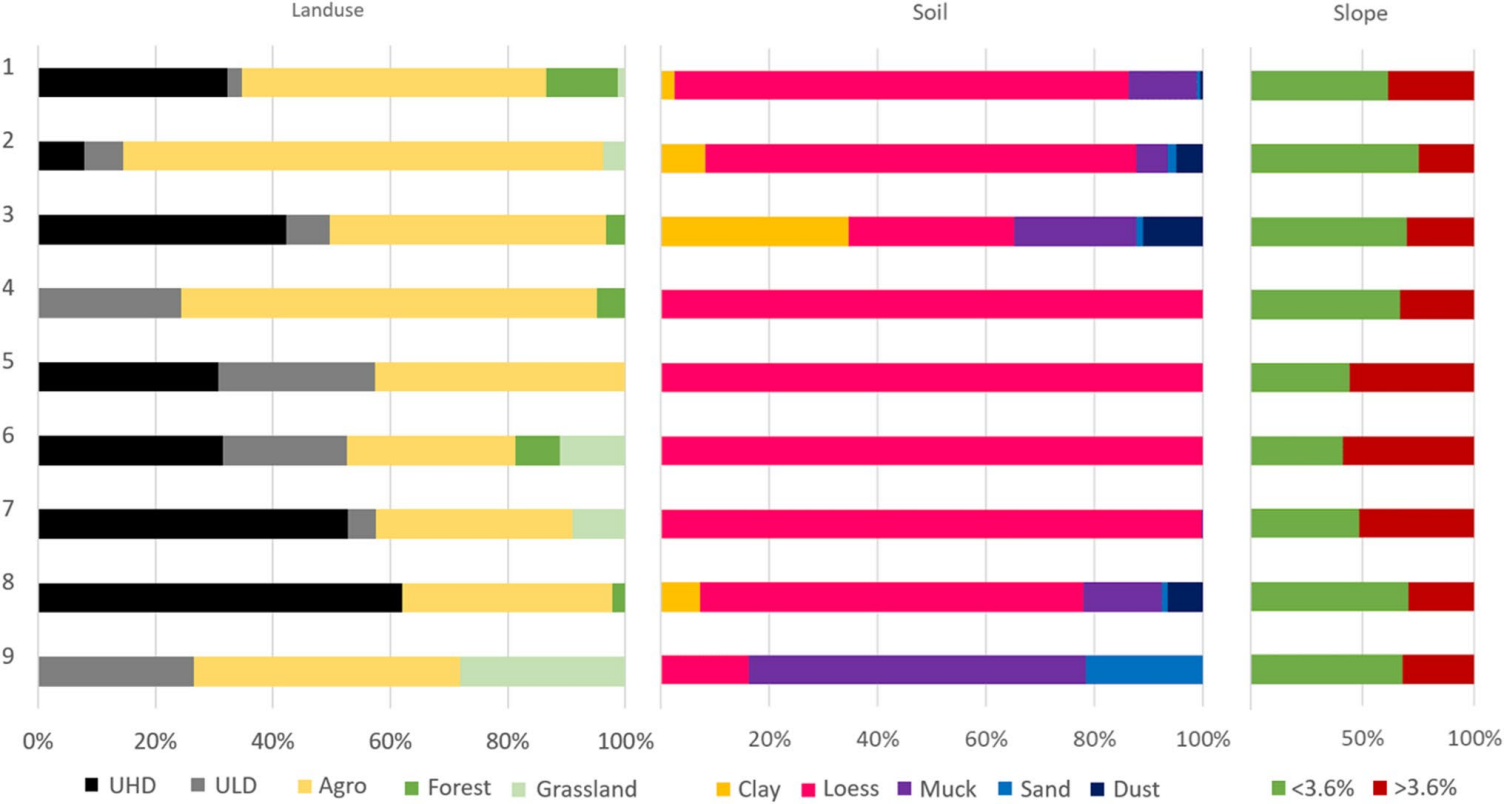
Land use, soil and slopes for the selected subbasins of the city of Lublin

The selected subbasins clearly differ in surface area, number of inhabitants (Table 1), topography and land use (Fig. 2). The Bystrzyca Lubelska River divides the city of Lublin area into two separate landscape parts (Kłosowski 2012; Zawardka 2018; MPA 2018). The left-bank part (subbasins 4, 5, 6 and 7 and partly 1, 8 and 9) is characterised by a varied topography with deep valleys and gorges. Consequently, the share of slopes > 3.6% is noticeably higher in this part (Fig. 2). The right-bank part (subbasins 2 and 3 and partly 1, 8 and 9) is definitely flatter and less topographically diverse, with a higher share of slopes < 3.6%.

**Table 1:** Surface area and number of inhabitants for the studied subbasins (Statistics Poland 2023)

Subbasin	Area (ha)	Approximate population (ths)
1	4716	78.7
2	2045	23.1
3	2130	41.1
4	1168	2.6
5	923	13.1
6	980	17.8
7	1234	27.0
8	2509	57.6
9	203	< 100 pers

The selected subbasins differ also in the share of impervious areas covered with high and low density of urban development. UHD occurs primarily in subbasins 1, 3, 7 and 8 (even over 50% of area), consisting mostly of the historic buildings of the Lublin Old Town and multi-family housing, as well as commercial, industrial, communication and institutional zones. Subbasins with the largest share of UHD are also characterised by the largest number of inhabitants (over 200,000 in total) (Table 1). ULD constitutes mainly single-family housing characterizing suburban areas in sub-basins 4, 5, 6 and 9, inhabited by a total of almost 34,000 people. UHD and ULD in the analysed subbasins are surrounded by other types of land use, such as agriculture, forests and grassland. Agricultural areas in the case of peripheral subbasin nos. 2 and 4 occupy about 80% of their area. Forest and grassland areas are concentrated in subbasins 1, 6 and 9, where their share reaches a maximum of 12% and 28%, respectively. The geological structure of the analysed area is dominated by loess cover, which in left-bank subbasins 4–7 constitutes even more than 70% of all soils. The remaining share is covered by clay, muck, sand and dusty soils (Fig. 2).

The city of Lublin is located in the zone of mixing influences of polar-marine air (66%) with continental air (20%). The average annual temperature is 7.9 ºC, with − 3.6 ºC in the coldest month (January) and + 18.6 ºC in the hottest month (July). The average annual precipitation reaches 566 mm. The prevailing winds are from the west and south-west, and their average speed is 2.5 m/s. The local climate conditions are closely related to the landform, course of river valleys and the existing urban development (SUiKZP 2019).

### Input data and model calibration

The digital platform Macromodel DNS/SWAT was developed at the Polish Institute of Meteorology and Water Management—National Research Institute (IMGW-PIB). The decisive factor in its use in the current research was the ability to combine the best features of models, methods and databases combined in one digital space, which has already been described in detail in Szalińska et al. (2020), Orlińska-Woźniak et al. (2021), Wilk et al. (2022) and Bojanowski et al. (2022). The most important module used in the Macromodel is the SWAT model, which is a physical, semi-distributed, continuous model running on a daily, monthly or yearly time step (Arnold et al. 2012). Its basic feature is the ability to continuously simulate hydrological and qualitative processes related to the transport of pollutants in both the land and river bed phases of the catchment (Kiros et al. 2015). The SWAT model allows for spatial differentiation of water balance elements. This tool divides a watershed into subbasins using a digital elevation model (DEM) (Table 2), and each subbasin is further divided into hydrologic response units (HRUs) based on a unique combination of land use, soil type and slope. In order to maintain a compromise between model accuracy and efficiency, land use has been simplified by excluding the numerous smallest HRUs while taking into account land use diversity. Model simulations have been performed at the HRU level, and the simulated outputs aggregated at the required subbasin level through routing processes (Lee et al. 2018). This division was used in the described research to separate nine subbasins of the Lublin agglomeration from the SWAT model for the Wieprz River catchment (Fig. 1), which was commissioned by the State Water Holding—Polish Waters (SWH-WP) under the project “Identification of pressures in water regions and river basin districts” (SWH-PW 2019).

**Table 2:** SWAT module input data, source and resolution for the Wieprz River basin area

Description	Data source	Data resolution
Map of Poland hydrographical divisions	State Water Holding-Polish Waters (PGW WP), https://isok.gov.pl/hydroportal.html	5 m, 1:10,000
Digital elevation model (DEM)	Nation Protection IT System, https://isok.gov.pl/index.html	10 m, 1:20,000
Cross-sections of river channels	Nation Protection IT System—https://isok.gov.pl/hydroportal.html	-
Land use map (development of the river banks)	Corine Land Cover (CLC 2012), images from the Landsat 8 satellite http://clc.gios.gov.pl/index.php/26-clc-2012, https://bdl.stat.gov.pl/BDL	10 m
Soil map	Institute of Soil Science and Plant Cultivation, forest soil maps	2.5 m, 1:100,000, 1: 10,000
Meteorological data—air temperature, precipitation, humidity, wind speed and total radiation	Institute of Meteorology and Water Management—National Research Institute (IMGW-PIB)—https://danepubliczne.imgw.pl/	For 250 stations located directly in the basin and within 20 km from its borders
Drained areas—average depth of drains, average drainage time	Chief Inspectorate for Environmental Protection—https://www.gios.gov.pl/pl/stan-srodowiska/monitoring-wod	-
Point sources of wastewater, population not connected to the sewage system and fish ponds	National Program for Urban Waste Water Treatment—https://www.wody.gov.pl/nasze-dzialania/krajowy-program-oczyszczania-sciekow-komunalnych, Central Statistical Office—https://bdl.stat.gov.pl/BDL, State Water Holding-Polish Waters	-
Agrotechnical treatments, livestock breeding, the amount of mineral and natural fertilizers	Central Statistical Office—https://bdl.stat.gov.pl/BDL/	-
Runoff from urbanized areas	Analysis of significant anthropogenic impacts’ database (PGW WP)	-
Atmospheric deposition, water erosion and geochemical background	Chief Inspectorate for Environmental Protection—http://powietrze.gios.gov.pl/pjp/archives	-

Development of the Wieprz River model required numerous point and nonpoint data at the appropriate level of detail, e.g. catchment topography, soil characteristics and distribution, land cover and meteorological conditions. The basic input data used to build this tool is presented in Table 2.

The Wieprz River model was calibrated, verified and validated to capture both aspects of simulation, quantitative (flow) and qualitative (total nitrogen (TN) and phosphorus (TP) and sediment). The choice of the time periods and calculation profiles was imposed by availability of the monitoring data. For the quantitative part, data on daily flows (source: IMGW-PIB) for the period of 15 years (2004–2018) from nine measuring stations located on the Wieprz River and its tributaries (Minina, Bystrzyca Lubelska and Łabuńka) were used. The data for the examination of the qualitative part of the SWAT (TN, TP and sediment) originated from five stations of the State Environmental Monitoring (SEM), located on the Wieprz River and its tributaries (Białka and Bystrzyca), covering a period of 14 years (2005–2018). The first 3 years (2004–2006) were used to condition the model (i.e. warm-up or run-up for the SWAT simulations) (Kulkarni et al. 2022) and the subsequent periods for calibration (2007–2011), verification (2012–2018—quantitative and qualitative model) and validation (2004–2015).

Calibration of the model was performed using the SWAT-CUP program developed by Abbaspour (2015) and the SUFI-2 algorithm. First, a visual assessment of the calibration quality was performed based on the 95 per cent prediction uncertainty (Nguyen et al. 2022) used for simulated and observed flows, sediment, TN and TP loads during the calibration, verification and validation period (Figs. SI1-6). Sensitivity analysis performed with the Latin Hypercube One factor-at-a-Time (LHOAT) sampling approach was used to identify the most influential estimation of constituent load (calibration) (Table SI7) (Khalid et al. 2016). Then, a formulated regression model was used to estimate loads over a user-specified time interval (estimation). Three statistical measures, Kling-Gupta efficiency (KGE) (Knoben et al. 2019), coefficient of determination (*R*^2^) (Zhang 2017) and per cent bias (PBIAS) (Pfannerstill et al. 2017), have been used to indicate the Wieprz River model performance, with the value ranges presented in Table SI8. For this model, the KGE coefficient was used as the main statistical measure, while the values of the *R*^2^ and PBIAS were monitored. This choice was dictated mainly by the fact that KGE led to hydrographs which better presented the regime of river flows of the studied river in visual assessment.

For the flow, KGE and PBIAS calibration and verification factors classified the model behaviour as generally very good and good for the main river (Wieprz) and its tributary (Tyśmiennica). Satisfactory and unsatisfactory values were characterised only by *R*^2^ coefficient. During the validation procedure, all values of the KGE and PBIAS coefficients rated the performance of the model for daily flow simulations as very good and good (Table SI9). In the case of quality parameters, a lower model performance was observed, from very good to unsatisfactory in the case of *R*^2^ (Table SI10). This was related to the high variability of the time distribution patterns for parameters, especially for TP and sediment. After successful completion of the calibration, verification and validation procedure and the introduction of parameter values giving the maximum value of statistical measures, such a simulation has been called the baseline scenario.

### Urban area runoff and nutrient load calculation methods

Despite taking into account different types of urban development (UHD and ULD) during the simulation, the SWAT model does not directly distinguish between these two types of urban land use in the result tables for the individual HRUs. This significantly limits the possibilities of analysing the response of both types of urban land use under the forecasted climate changes. In order to overcome this problem, a method was proposed to distinguish between UHD and ULD using typical hydrological parameters. We hypothesised that the two model parameters, PERC and SURQ, will effectively classify HRU from the selected subbasins into the appropriate UHD or ULD groups. PERC represents the percolation process, i.e. redistribution of infiltrated water in the soil profile, predicting flow through each soil layer (Mapes and Pricope 2020). On the other hand, SURQ is one of the components of water yield (Sakizadeh et al. 2023), representing surface runoff which depends on the sum of precipitation, evaporation and soil water storage (Zang et al. 2019; Mengistu et al. 2022).

PERC and SURQ for UHD and UHL in the SWAT model have been calculated for each HRU based on the Soil Conservation Service (SCS) curve number (CN) method (Neitsch et al. 2011) developed to simulate surface runoff from daily rainfall events, which assumes that water from surface runoff is delivered to streams or further percolated into groundwater. The groundwater portion is then transported to streams through groundwater flow, percolated into the deep groundwater aquifer or discharged to the soil profile (Lee et al. 2018). This method first estimates the amount of surface runoff and assumes that the remaining precipitation will infiltrate (Zhang et al. 2019). Surface runoff is defined as Eq. 1:

1 $${Q}_{\text{surf}}={\left({P}_{i}-{I}_{a}\right)}^{2}/\left({P}_{i}-{I}_{a}+S\right)$$

where *Q*_surf_ is the surface runoff or rainfall excess (mm H_2_O); *P*_*i*_ is the precipitation depth for the day (mm H_2_O); *I*_*a*_ is the initial abstraction lost from canopy interception, surface storage and infiltration prior to runoff (mm H_2_O) and *S* is the retention parameter (mm H_2_O), which is estimated by following Eq. 2:

2 $$S=25.4\left(1000/CN -10\right)$$

where *CN* is the curve number value for the day, which is a function of the soil permeability, land use and precedent soil water content.

Subsequently, the load of pollutants transported with surface runoff from the urbanized areas has been calculated with the use of the build-up/wash off component of the SWAT model. In this option, sediment particles with contaminants are collected on impervious surfaces (land phase) on dry days (build up), and then washed away with the appearance of precipitation to finally end up in the river bed phase (wash off).

In the SWAT model, impervious areas can be differentiated into two groups: areas mostly hydraulically connected to the drainage system—UHD and areas mostly not connected directly to this system—ULD. Therefore, surface runoff is calculated separately for both types of areas. For UHD, the curve number 98 (Kumar et al. 2021) is always used, while for ULD, a composite curve number is calculated and used. The equation used to calculate the composite curve number for disconnected impervious areas is Eq. 3:

3 $${CN}_{c}=\left\{\begin{array}{c}{CN}_{p}+{imp}_{\text{tot}}*\left({CN}_{\text{imp}}-{CN}_{p}\right)*\left(1-{imp}_{\text{dcon}}/2* {imp}_{\text{tot}}\right) if\; {imp}_{\text{tot}}<0.30\\ {CN}_{p}+{imp}_{\text{tot}}*\left({CN}_{\text{imp}}-{CN}_{p}\right) if\; {imp}_{\text{tot} }>0.30\end{array}\right.$$

where *CN*_*c*_ is the composite moisture condition II curve number, *CN*_*p*_ is the pervious moisture condition II curve number, *CN*_imp_ is the impervious moisture condition II curve number, *imp*_tot_ is the fraction of the HRU area that is impervious and *imp*_dcon_ is the fraction of the HRU area that is impervious, but not hydraulically connected to the drainage system.

The fraction of the HRU area that is impervious but not hydraulically connected to the drainage system (*imp*_dcon_) is calculated according to the following Eq. 4:

4 $${imp}_{\text{dcon}}= {imp}_{\text{tot}}-{imp}_{\text{con}}$$

where *imp*_*t*ot_ is the fraction of the HRU area that is impervious (connected and disconnected), and *imp*_con_ is the fraction of the HRU area that is impervious and hydraulically connected to the drainage system.

From the impervious share of the HRU, the model uses the build-up/wash off algorithm to determine sediment and nutrient loadings. The build-up/wash off algorithm calculates the accumulation and wash off solids. This algorithm uses organic and mineral nitrogen and phosphorus concentrations, included in the model as default values, depending on area function of urban land type. Therefore, total nitrogen and phosphorus loads (TN and TP) in the impervious portion of the urban area are calculated by multiplying their concentration by the sediment loading. For the pervious share of the urban area (agro, forest and grassland), the MUSLE method is used to estimate sediment loads (Gwapedza et al. 2018). In the case of TN and TP, the model simulates nutrient cycling, i.e. the transport and transformation of many forms of nitrogen and phosphorus in water and soil (Krysanova and Srinivasan 2015; Zeiger and Hubbart 2016). The final results of the simulations are produced by the SWAT module as the forms of nitrogen and phosphorus are then summed up to give TN and TP values.

### Climate scenarios

The climate scenarios have been developed using the Urban Adaptation Plans (UAP) project (https://www.gov.pl/web/klimat/mpa-44) predictions, based on the data from the Euro-CORDEX, regional climate models (RCM) (Rummukainen 2016; Dosio 2016) and the global climate models (GCM) (Wootten et al. 2017). The GCM-RCM model pairs used in the current study are shown in Table SI11. Before being effectively used, the RCMs underwent bias correction using the quantile mapping approach (Holthuijzen et al. 2022; Rajulapati and Papalexiou 2023). The selection of the final model ensemble for this particular area was performed based on the skill to simulate present and near-past climate or the ability to represent the same connection pattern that drives the climate of the studied region (Lutz et al. 2016; Khan and Koch 2018).

Data from the Lublin synoptic station (51.218361, 22.393142), located 12 km away from the centre of the agglomeration, has been used. The statistical postprocessing (downscaling) (Luo 2016; Iturbide et al. 2019) was performed using the change factor method (Lanzante et al. 2020; Tabari et al. 2021). The climate condition analysis in the UAP project covered the moderate (RCP4.5) and extrapolative (RCP8.5) scenarios and two future time horizons covering a short-term perspective (average of 2026–2035) and a long-term perspective (average of 2046–2055) (Dobler et al. 2018). Therefore, four climate variant scenarios, with a monthly time step, were prepared to combine the RCP predictions and adopted time horizons: RCP4.5 (2026–2035)—VS1, RCP4.5 (2046–2055)—VS2, RCP8.5 (2026–2035)—VS3 and RCP8.5 (2046–2055)—VS4.

**Fig. 3:**
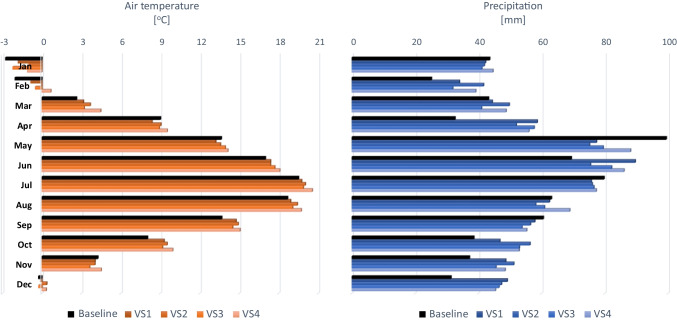
Average monthly precipitation and temperature for the baseline and climate change scenarios for the city of Lublin

All the scenarios used in this study indicated that significant changes in both temperature and precipitation should be expected in both the short- and long-term scenarios. For both extrapolation scenarios (VS3 and VS4), the largest changes in relation to the baseline scenario should be expected in the autumn and winter months, where the temperature increase is forecast to be as much as 1.9–2.7 °C in October and February, respectively (Fig. 3). On the other hand, April will be the month characterised by the largest average decrease in temperature in both time horizons (even by 0.6 °C in VS1). A similar pattern of change can also be expected for the moderate scenarios (VS1 and VS2). The dynamics of change in the case of precipitation will be even more pronounced. In this case, the moderate scenarios show the greatest changes in February, April, June, October, November and December, when the monthly average values can increase by more than 26 mm (VS1) (Fig. 3). In turn, the largest drop in precipitation is expected in May, reaching over 20 mm in VS2.

## Results

### Separation of UHD and ULD

In the selected nine subbasins covering the city of Lublin, the different number of HRUs classified as representing urban development has been detected (1–7). As hypothesised in “Urban area runoff and nutrient load calculation methods,” PERC and SURQ have been used to distinguish between high- and low-density developments. Division of HRUs and subsequent separation of UHD and ULD has been performed with the use of cluster analysis, based on the sum of squared Euclidean distance, performed with the STATGRAPHICS 19 software. The PERC and SURQ characteristic values for both types of urban development in each subbasin have been presented in Table 3. Generally, an average (AV) with coefficient of variation (CV) has been given with some exceptions due to the insufficient amount of data, marked with an asterisk. The PERC values in subbasins 5, 6 and 7 equal 0.00, resulting from the 100% impervious surface covering HRUs and preventing the percolation process, have been classified as UHD.

**Table 3:** PERC and SURQ (mm) characteristic values for the selected subbasins within the city of Lublin

Subbasin	Average of PERCmm				Average of SURQ_GENmm			
	UHD		ULD		UHD		ULD	
	AV	CV	AV	CV	AV	CV	AV	CV
1	0.86	122.9%	3.25^a^	-	14.34	6.0%	4.95^a^	-
2	0.67	141.4%	4.36	31.9%	14.35	6.3%	5.2	7.7%
3	0.64	141.7%	2.97^a^	-	14.6	6.8%	4.80^a^	-
4	-	-	3.56	1.4%	-	-	5.03	0.2%
5	0.00	-	2.95	1.7%	14.15	0.2%	4.85	0.1%
6	0.00	-	2.97	1.3%	14.16	0.1%	4.85	0.1%
7	0.00	-	2.98^a^	-	14.05	0.2%	4.81^a^	-
8	0.56	141.9%	-	-	14.13	5.9%	-	-
9	-	-	8.07^a^	-	-	-	4.35^a^	-
All	0.52	165.2%	3.75	40.2%	14.29	5.7%	4.9	5.5%

^a^Values different than average and coefficient of variation, AV and CV

Both parameters displayed significantly different values (Kolmogorov–Smirnov at *p* < 0.05) for UHD and UHL. Low values of PERC (0.00–0.86 mm) and high values of SURQ (14.05–14.60 mm) have been observed for UHD and high values of PERC (2.97–8.07 mm) and low values of SURQ (4.35–5.03 mm) for ULD. The adopted apportionment UHD and ULD has been used subsequently to discuss TN and TP loads from the city of Lublin.

### Nutrient loads

TN and TP loads discharged with the surface runoff from the entire city of Lublin area to the Bystrzyca Lubelska River simulated in the baseline scenario reached 56.8 t/year (tons per year) of TN and 3.8 t/year of TP (Table 4). The results showed a significantly higher contribution of TN and TP from UHD compared to ULD by 32,022 kg/year (86%) and 2574 kg/year (89%), respectively.Among the other land use types, agriculture contributed almost 95% of the remaining TN and TP loads. Since the studied area has been divided into nine subbasins, their individual TN and TP loads have been also provided by the baseline simulation. For TN, the loads varied greatly from 15.7 t/year for subbasin 7 to 0.4 t/year for subbasin 9. The same subbasins were also distinguished by the highest and lowest TP loads, i.e. 1.0 and 0.02 t/year for the subbasins 7 and 9, respectively.

**Table 4:** Average TN and TP loads in the surface runoff from the selected subbasins within the city of Lublin (kg/year and kg/ha/year) in the baseline scenario

	Subbasin\Land	Impervious area				Pervious area						SUM
		UHD		ULD		Agro		Forest		Grassland		
	use	kg/y	kg/ha/y	kg/y	kg/ha/y	kg/y	kg/ha/y	kg/y	kg/ha/y	kg/y	kg/ha/y	kg/y
TN	1	10 188	6.70	436	3.84	4 314	1.77	617	1.06	114	1.95	15 670
	2	1 150	7.25	529	4.05	2 632	1.61	-	-	143	1.91	4 454
	3	6 119	6.93	603	3.90	1 871	1.90	106	1.23	-	-	8 699
	4	-	-	1 164	4.38	1 293	1.67	37	0.49	-	-	2 494
	5	2 080	7.57	984	4.13	609	1.60	-	-	-	-	3 672
	6	2 227	7.43	815	4.05	423	1.55	39	0.39	128	1.20	3 631
	7	4 596	7.17	216	3.72	626	1.54	-	-	132	1.20	5 570
	8	10 617	7.08	-	-	1 503	1.73	117	1.19	-	-	12 238
	9	-	-	209	3.91	98	1.07	-	-	105	1.84	411
	sum	36 978	-	4 956	-	13 368	-	916	-	621	-	56 839
TP	1	786	0.52	25	0.22	187	0.08	22	0.04	4	0.07	1024
	2	89	0.56	31	0.24	116	0.07	-	-	5	0.07	241
	3	470	0.53	35	0.23	89	0.09	4	0.04	-	-	597
	4	-	-	73	0.28	59	0.08	1	0.02	-	-	134
	5	165	0.60	61	0.26	30	0.08	-	-	-	-	255
	6	171	0.57	50	0.25	20	0.07	2	0.02	5	0.05	247
	7	362	0.57	13	0.22	29	0.07	-	-	5	0.05	410
	8	832	0.55	-	-	73	0.08	4	0.04	-	-	909
	9	-	-	12	0.22	5	0.06	-	-	4	0.07	21
	Sum	2874	-	300	-	608	-	34	-	23	-	3838

Although such vast differences in nutrient loads have been caused mainly by differences in the surface area of particular subbasins, and their internal land use distribution, the differences in unit loads should also be noticed (Table 4), especially when both types of urban development are taken into consideration, with the values for UHD (7.16 and 0.56 kg/ha/year for TN and TP, respectively) almost double the size compared to ULD (3.99 and 0.24 kg/ha/year for TN and TP, respectively). As for the remaining land use types, the unit load variability among the areas located in the right and left-bank Bystrzyca Lubelska River subbasins should be observed. Higher unit loads for TN and TP have been noticed for agriculture and forest in subbasins 3 and 8 than in subbasins 4 and 6, which is most likely related to the different soil types characterizing both river banks.

**Fig. 4:**
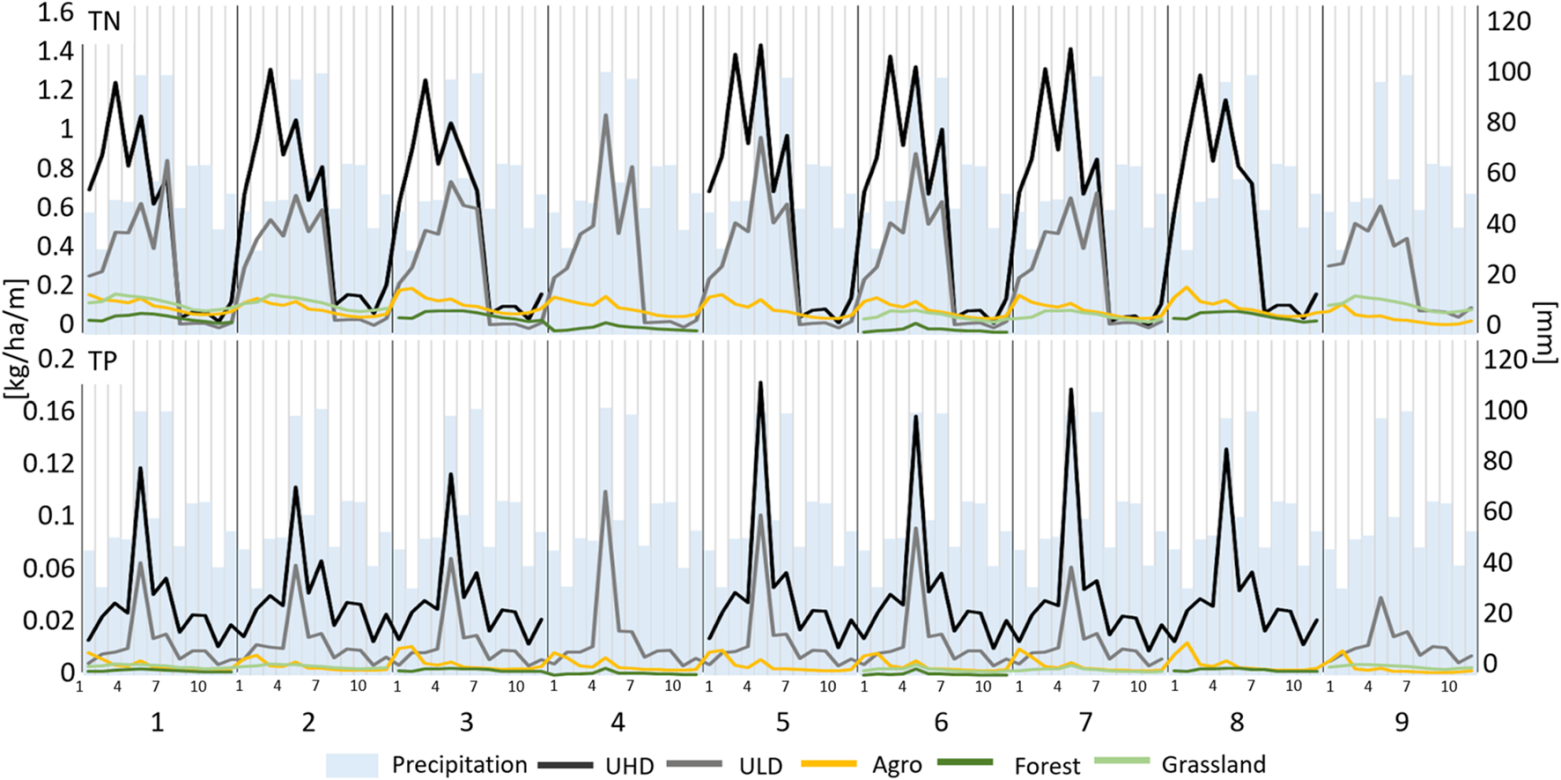
Monthly distribution of the TN and TP unit loads in the surface runoff from the city of Lublin subbasins with an average monthly precipitation

The difference between both types of urban and remaining sources is also clearly visible when monthly load distribution is taken into consideration (Fig. 4). Apart from the obvious disparity in monthly loads, a differing pattern of nutrient discharges should be observed. For both urban types of TN sources, elevated loads have been observed in March (up to 1.4 kg/ha/month, kg/ha/m, in subbasin 5) with the increase of snowmelt and in May and July (up to 1.42 kg/ha/m in subbasins 5 and 7) when the amount of precipitation is distinctly increasing. For the agricultural source, an initial load increase is noticeable in February (up to 0.24 kg/ha/m in subbasins 3 and 8) when the surface runoff is facilitated by the lack of vegetation cover, and nitrogen is also sourced from the early fertilization practices. The same pattern for the agricultural source is also visible for TP loads, reaching up to 0.02 kg/ha/m. While for UHD and ULD, a distinct increase is noticed along with the May precipitation increase with the loads at a level of 0.15–0.17 kg/ha/m.

### Climate change impact on urban nutrient loads

The distribution of changes in TN and TP loads (Table 5), resulting from the variant scenarios, generally shows an increase in pollutant outflow from all sources compared to the baseline scenario. However, the response of individual sources will be significantly different depending on the type of land use and the time horizon. For impervious areas, UHD and ULD, the average increase in total and unit nitrogen loads will be about 12% (3600 and 700 kg/year and 0.73 and 0.6 kg/ha/year, respectively) and for phosphorus about 18% (515 and 49 kg/year and 0.09 and 0.04 kg/ha/year, respectively). The most distinct reaction to the changes should be expected in the VS1 scenario, where the total average outflow of TN and TP will increase by as much as 17% (5213 kg N/year and 581 kg P/year) and almost 19% (911 kg N/year and 58 kg P/year) from UHD and ULD, respectively (Table 5).

**Table 5:** Average annual TN and TP loads in surface runoff in the city of Lublin (kg/year and kg/ha/year) in the baseline and variant scenarios (VS1–VS4)

	Scenario\Land use	Impervious area				Pervious area						SUM
		UHD		ULD		Agro		Forest		Grassland		
		kg/y	kg/ha/y	kg/y	kg/ha/y	kg/y	kg/ha/y	kg/y	kg/ha/y	kg/y	kg/ha/y	kg/y
TN	Base	36,978	7.16	4956	4.00	13,368	1.61	916	0.87	621	1.62	56,839
	VS1	42,190	8.2	5868	4.76	20,868	2.52	1740	1.75	992	2.53	71,657
	VS2	40,893	7.97	5679	4.64	21,248	2.56	1807	1.81	1025	2.61	70,651
	VS3	39,785	7.72	5549	4.51	19,533	2.36	1573	1.56	922	2.36	67,363
	VS4	39,598	7.68	5516	4.51	21,911	2.63	1809	1.81	1030	2.63	69,863
TP	Base	2874	0.56	300	0.24	608	0.08	34	0.03	23	0.06	3838
	VS1	3454	0.67	359	0.29	925	0.12	63	0.06	36	0.09	4837
	VS2	3381	0.65	337	0.27	885	0.11	65	0.07	37	0.09	4705
	VS3	3309	0.64	343	0.28	854	0.11	57	0.06	33	0.09	4595
	VS4	3414	0.66	361	0.29	927	0.11	66	0.07	37	0.09	4804

The average increase in loads from sources located in the pervious areas will be even greater and will amount to about 60% for agriculture (over 7500 kg N/year and 335 kg P/year, respectively) and grassland (30 kg N/year and almost 14 kg P/year, respectively) (Table 6). However, the greatest reactivity to climate change should be expected in forests, where the average increase in nutrient outflow will be as much as 90% (815 kg N/y and 29 kg P/y, respectively). Average changes in unit nutrient loads for each of these sources will be similar and amount to 0.9 kg N/ha/y and 0.03 kg P/ha/y (Table SI12). Sources located on pervious areas will show the most significant reactivity in the long time period of the VS4 scenario. Therefore, an average increase in agriculture and grassland loads by about 65% (8500 kg/y and 400 kg/y, respectively) and from forests by up to 97% (nearly 900 kg/y) should be expected in this scenario (Table 6).

**Table 6:**
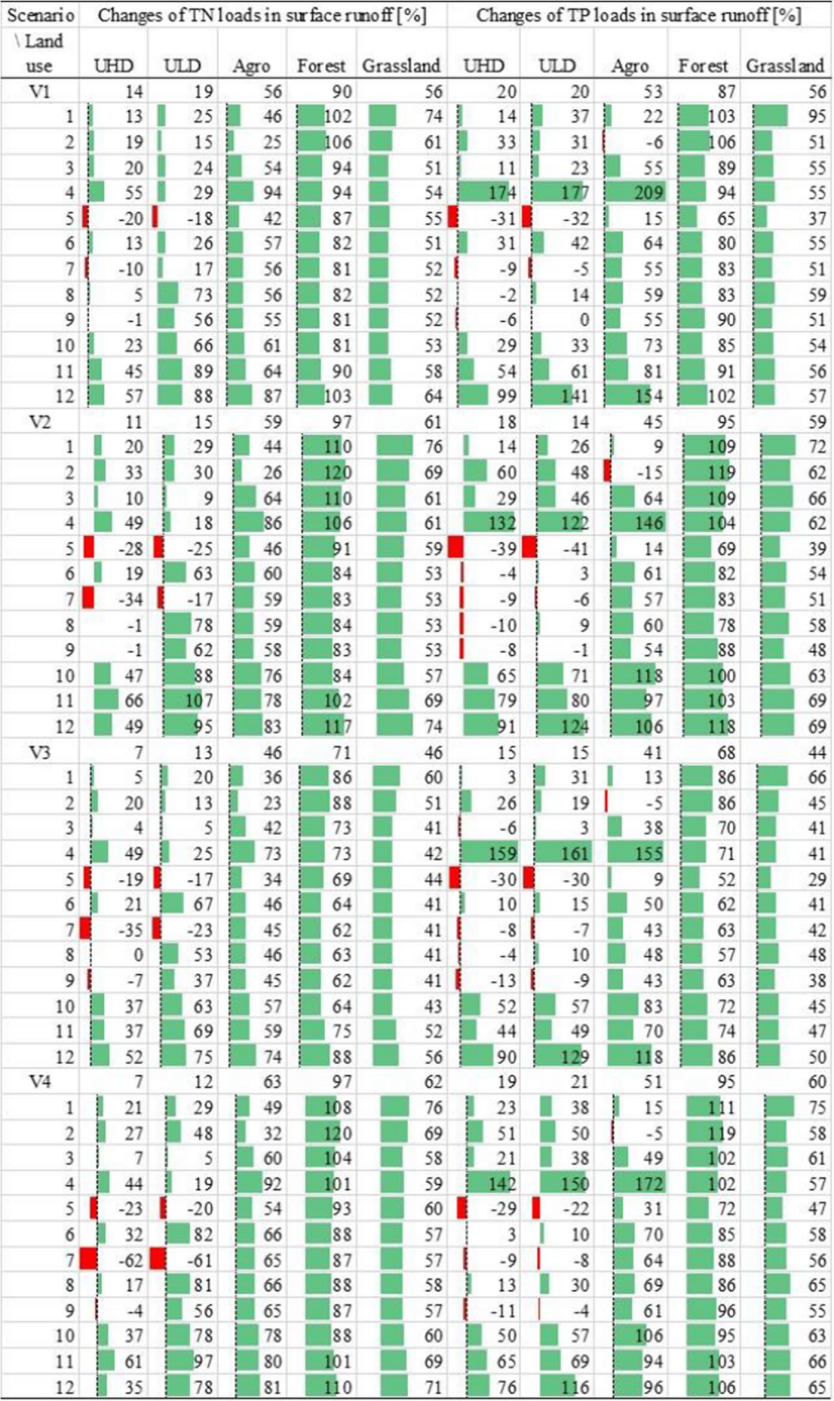
Changes of TN and TP loads (%) in surface runoff in the city of Lublin in variant scenarios (VS1–VS4)

The analysis of temporal changes made it also possible to identify months in which pronounced changes in nutrient loads can be expected in relation to the baseline scenario. In this context, the increase of nutrient outflows from impervious areas in April is particularly noteworthy. In this month, a significant increase in total and unit loads of TN by up to 55% (2500 kg/m and 0.47 kg/ha/m for UHD) and for TP more than 177% (37 kg/m and 0.031 kg/ha/m) should be expected for ULD in the VS1 scenario (Tables 6 and SI5). The following months, especially May and July, show a reduction of the TN and TP outflow from both urban sources by as much as 60% and 40%, respectively. During the rest of the year, differences in TN load outflow between both types of urban areas are visible in all variant scenarios. While an increase from UHD reaching up to 66% (286 kg/m and 0.06 kg/ha/m) can be expected only in October–December, for ULD, these changes start from August. As for TP, such monthly differences are not visible, and the maximum changes reach 99% and 141% (175 kg/m and 0.03 kg/ha/m and 18 kg/m and 0.01 kg/ha/m for UHD and ULD, respectively) (Tables 6 and SI5).

## Discussion

The methodology shown in our article allowed us to effectively separate two types of urban impervious surfaces, characterised by high and low density of development (UHD and ULD). To perform this separation, we have used a contrasting behaviour of percolation and surface runoff values for each HRU unit, described by two hydrological parameters delivered by the Macromodel DNS/SWAT simulations (PERC and SURQ). In case of UHD, almost all vegetation has been replaced by impervious areas; therefore, the surface runoff transports accumulated sediments along with nutrients directly to the drainage system (Jiang et al. 2015; Russell et al. 2017; Seo et al. 2017; Ferreira et al. 2018; Gorgoglione et al. 2019; Strohbach et al. 2019; Wang et al. 2021). The nature of the ULD is more varied and although it is still an urban area, a higher share of pervious surfaces (e.g. lawns and gardens) facilitates percolation of rainwater and limits the loss of nutrients (Flörke et al. 2018). Nitrogen in the urban areas results mainly from commercial, municipal or industrial activities, as well as household pet waste and usage of fertilizers in urban gardens (Zhang et al. 2016; Winiwarter et al. 2020; Kaltenegger et al. 2023; Sieczko et al. 2023), which in Lublin are located even in the city centre. In turn, the most important sources of phosphorus are atmospheric deposition and weathering, especially from arable land surrounding this city (Song et al. 2017; Hobbie et al. 2017; Bittman et al. 2017; Yang and Toor 2018; Small et al. 2019).

The release of TN and TP loads from agricultural, forest and grassland sources located in the city of Lublin is mainly controlled by factors such as slope size, type of soil and land use. Therefore, a visible differentiation of unit loads of nutrients between the sources located on the right and left banks of the Bystrzyca Lubelska River, which differ significantly in topography and soil types (Mroczek 2014; Sowińska-Świerkosz et al. 2021), has been observed. Nevertheless, the UHD and ULD surface, occupying on average 28% and 13% of the studied area, exceeds the critical value for an impervious surface (10%) (Yang and Lusk 2018), which indicates the possibility of noticeable degradation of the quality of surface runoff from this area. Indeed, our baseline scenario results showed a dominant share (approx. 80%) of combined UHD and ULD sources for both TN and TP. However, unit nutrient loads indicated almost twice as large an outflow of pollutants from UHD than from ULD (Table 5). As mentioned before, absence of the percolation process from the impervious UHD surfaces enhances surface runoff and is responsible for elevated TN and TP loads. While in ULD areas, constituting a transitional/hybrid form between impervious and pervious surfaces, percolation and vegetation decrease nutrient loss.

The difference between UHD-ULD and the other sources is also clearly visible in the monthly nutrient load distribution (Fig. 4). The variability of loads from urban sources is related directly to meteorological phenomena, which is considered to be the main transport vector for pollutant release in the urban environment (Glińska-Lewczuk et al. 2016; Yang and Toor 2017; Müller et al. 2020). This is reflected in the simulation results, where the increase of TN and TP loads in early spring (February–March) is mainly caused by the melting of snow cover (Janicki 2014). While an increase in the May–July period is caused by high precipitation (approx. 100 mm on average) (Fig. 4), these months are considered the rainiest period of the year in Poland (Kubiak-Wójcicka 2020). As for the nutrient discharges from agriculture, the largest increase in loads is observed already in February (Fig. 4), when intensive fertilization of arable land begins after the winter break (Panagos et al. 2015) and on soils still devoid of vegetation. On the other hand, loads from this source are much less reactive to high rainfalls in spring and summer than in urban areas (Fig. 4).

The implementation of the climate variant scenarios generally indicated an increase of nutrient outflow in relation to the baseline scenario from all subbasins and land use types of the city of Lublin. The forest responded best to the variant scenarios. Such a response, reaching over 100% (approx. 900 kg N/year and 30 kg P/year) of the base scenario values for both TN and TP has also been observed for the other basins in Poland (Bojanowski et al. 2023). While for the remaining sources, agriculture and grassland, these differences remained at a similar level (44–60%) as that of the baseline scenario level. As for the differences among the variant scenarios, it should be noticed that the long-term scenarios (VS2 and VS4) will generate higher nutrient loads then their short-term counterparts (VS1 and VS3), which is related to higher precipitation predicted in these scenarios. In turn, for both types of urban areas, this increase will reach even about 20%, which means that nutrient loads may increase by approx. 6000 kg/year and 500 kg/year for TN and TP, respectively. This confirms that urban land use will remain the largest source of pollution in the analysed catchment. Moreover, the difference in unit loads between TN and TP for UHD and ULD will also remain an important feature differentiating both types of urban sources. The differences between average UHD and ULD unit loads for the whole studied area, up to 5% for both TN and TP, become noticeably larger when results are discussed at the subbasin level. For the selected subbasins, this difference reached up to 11% and 13% for TN and TP, respectively, in scenario VS3 and VS4. The reasons behind this stronger response to the variant scenarios, especially in subbasins 1 and 7, seem to be related to the location of these areas on the steeper bank of the Bystrzyca Lubelska River, forcing outflow from pervious areas of these subbasins.

**Fig. 5:**
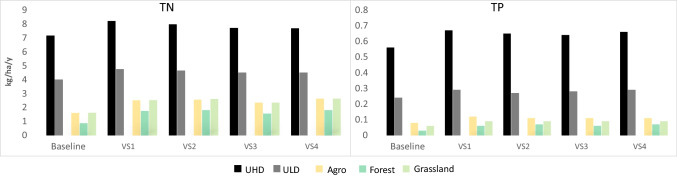
Average nitrogen and total phosphorus unit loads in the VS1–VS4 scenarios in relation to the baseline scenario

**Fig. 6:**
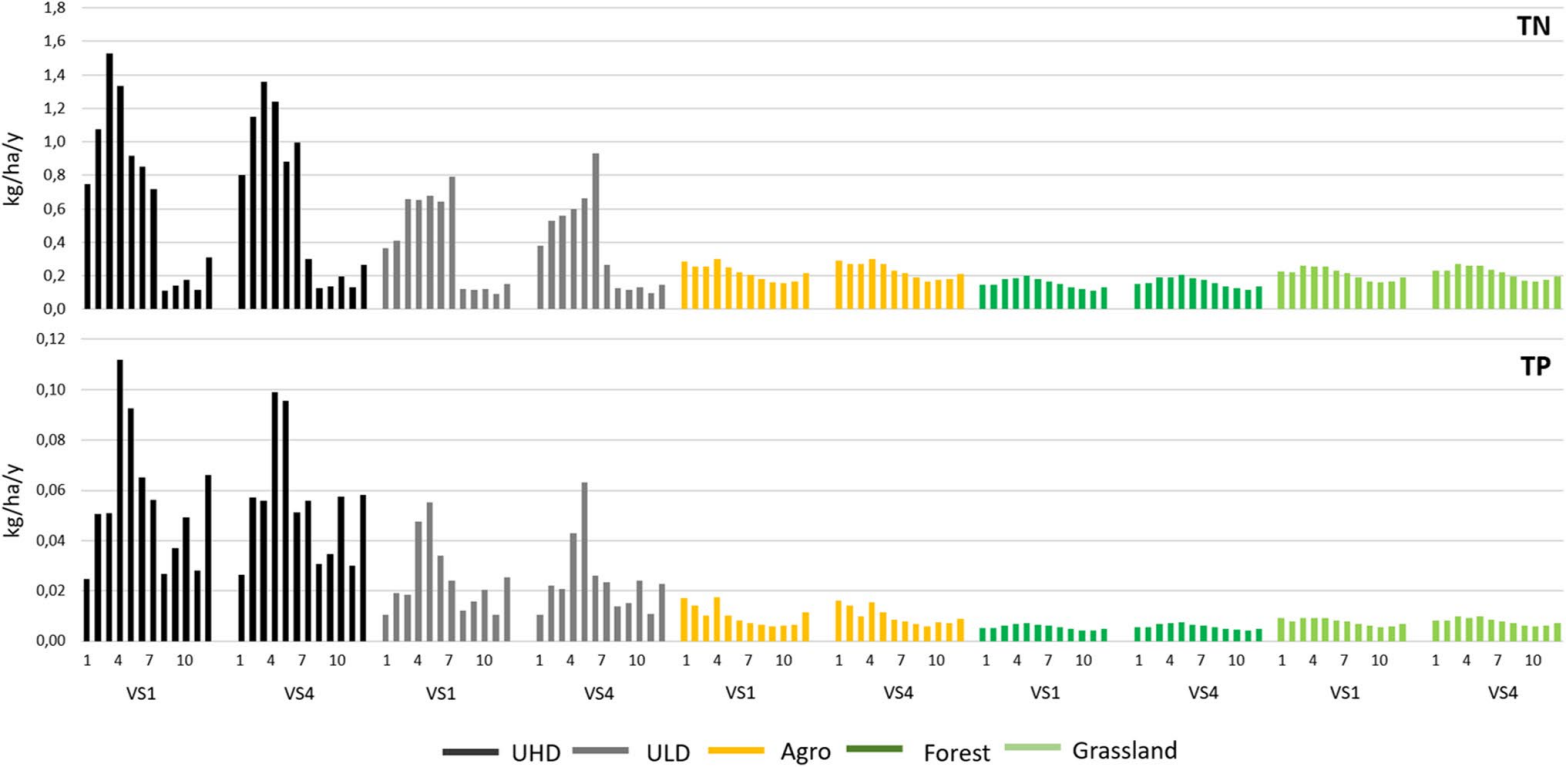
Monthly unit loads of nitrogen and total phosphorus in selected variant scenarios (VS1 and VS4)

By analysing the impact of climate change on monthly load distribution, we were also able to check whether TN and TP would remain effective differentiators between the two types of urban development. In the case of TN, a similar pattern of behaviour of unit loads for both UHD and ULD should be expected over the course of the year. Clear division of the year into two parts, already observed in the baseline scenario (Fig. 5), will be maintained as shown in the example of the two selected variant scenarios, VS1 and VS4 (Fig. 6). The size of TN loads flowing from both types of urban development in the first half of the year distinguishes UHD and ULD more clearly (Fig. 6). It is expected that the already high TN load outflow from the UHD in spring (up to 1.27 kg N/ha/m and 0.83 kg P/ha/m) will increase further, especially in April (by up to 55%), as a consequence of the forecasted growth in precipitation (Fig. 3). The second half of the year, in turn, means a much greater similarity of TN unit loads outflowing from UHD and ULD. This seems to be related to significantly lower precipitation in this period, and also to differences in nitrogen mobility in fully or partially impervious areas (Hobbie et al. 2017; Zhang et al. 2021). The monthly time step of the analyses also proved a greater variability of TP when compared with TN. It results primarily from the effective mobilization of phosphorus through impervious areas that prevent its particles from being trapped by vegetation and soil and preventing their infiltration (Hobbie et al. 2017; Kincaid et al. 2020). As a result, the urban mobility of phosphorus contributes to increasing its reactivity in the variant scenarios, which is particularly visible in the spring and autumn–winter periods for unit loads (Fig. 6). Additionally, unlike nitrogen, there is a clear difference in the size of TP unit loads between UHD and ULD in almost all months of the year. For the remaining sources representing pervious areas (agriculture, forest and grassland), a much lower diversity of monthly unit loads is expected then for UHD and ULD (Fig. 6). Also in this case, a greater outflow of nutrients should be expected mainly in the spring and summer months; however, this difference is not very distinct and does not allow distinguishing between these sources as easily as for UHD and ULD.

It should also be noticed that nutrient loads from UHD and ULD in the future time horizons, as predicted under variant scenarios, could be additionally increased by the urban area expansion induced by the war in Ukraine. Already during the period of 2000–2018, the city of Lublin recorded a dynamic expansion of urban development with an increase of ULD areas by 13% at the expense of agriculture (Adamska 2021). Further changes are expected due to the mass migration of Ukrainian refugees. The analysis presented in the recent report (Wojdat and Cywiński 2022) shows that Lublin, as the largest city in the eastern part of the country, quickly grew by hundreds of thousands of new residents. It is assumed that due to the relatively lower cost of living compared to the western part of the country, and the attractiveness of this region, Lublin will remain one of the most important migration destinations. This poses new challenges for local government policy, primarily related to single and multi-family housing (Wodzicki et al. 2022), to provide residents and refugees with temporary and permanent accommodation. To estimate approximately the possible increase of urban areas, and the resulting increase of nutrient loads, the following data have been used: (i) the latest forecasts about the current and future housing demand (Wodzicki et al. 2022); (ii) the migration rate after the start of the war in Ukraine (Wojdat and Cywiński 2022); (iii) the number of people per apartment (Statistics Poland 2023) and (iv) the average area occupied by single and multi-family housing (Dąbrowska-Milewska 2010). As a result, we estimated the increase in UHD and ULD areas necessary to meet the long-term housing needs of Lublin at a level of approx. 160 and 360 ha, respectively. Therefore, the pressure exerted by both types of urban development on the nutrient loads will increase by over 5600 kg N/year and 380 kg P/year, respectively (13% and 12%).

Our results provide a quantitative assessment of nutrient loads attributed into two types of urban development sources and expected changes under climate change scenarios. Therefore, they should draw attention not just of environmental modellers but above all decision-makers responsible for further development of urban areas and condition of surface waters in their surroundings. Our results emphasize an urgent need for proper planning and implementation of mitigation strategies and management practices to prevent the predicted increase of nutrient loads released from urban areas (Miller and Hutchins 2017). These activities require a multi-faceted approach that integrates green infrastructure, urban planning and community engagement (Liu et al. 2016, 2017; Bixler et al. 2019). Facilities such as urban gardens and green roofs can effectively capture, absorb and filter surface runoff, retaining the nutrient load (Gong et al. 2020; Karabay et al. 2024). Proper management of rainwater is also important since detention basins can effectively retain rainwater, allowing sediment and nutrients to settle (Teurlincx et al. 2019). In parallel, supporting elements such as public education and nutrient management plans with guidelines on the fertilizer use in urban landscape should be implemented. The final element is the implementation and enforcement of more stringent regulations limiting nutrient discharges from both point and nonpoint sources, combined with programs to encourage property owners to reduce their nutrient footprint.

Although the digital platform Macromodel DNS/SWAT turned out to be very useful in estimating the outflow of nutrient loads from urban areas, and the developed method allowed differentiating between UHD and ULD, possible limitations should be kept in mind. In general, the land phase simulation in SWAT, crucial for differentiating between pervious and impervious areas, is not disputed by the scientific community (Sarkar et al. 2019). Nevertheless, due to the semi-empirical nature of this model, the impact of the adopted computational equations and the corresponding coefficients (e.g. the build-up/wash off component) may be a potential source of bias. This is confirmed by the preliminary results of in situ tests, which showed that for the city of Lublin, the concentrations of nutrients on sediment particles are noticeably higher when compared to the default SWAT values. Therefore, the presented results are potentially underestimated. Also, the default Soil Conservation Service Curve Number (SCS-CN) method (Ghoraba 2015), which takes into account at most the daily time step of precipitation occurrence, is considered oversimplified for cities where impervious surfaces dominate. For such areas, it is important to be able to take into account more accurate monitoring data reflecting the occurrence of precipitation events, especially short-term and intense ones. Therefore, in further research, it is planned to use the Green and Ampt method (Mao et al. 2016) which permits the use of rainfall data with a time step of 15 min. It should be also noted that not only does the SWAT model have its limitations, but also climate scenarios by their nature are burdened with significant uncertainty. This applies to both global and regional climate models; therefore, it is important to be aware of the wide range of possible results and projection biases. Currently, available forecasts are usually characterised by a large spatial scale and low temporal resolution (Fatichi et al. 2016). Therefore, their application to catchments with limited extreme weather areas should only be used as a general indication of expected changes. It should be also remembered that the final composition of the model ensemble needs to be analysed in terms of wet and dry projections, since it is of particular meaning for environmental modellers dealing with pollutants transported from the catchment.

## Conclusions

In the current study, we demonstrated the dominant impact of urban land use on nutrient loads outflowing in the surface runoff from a basin occupied by a big city. This outflow drastically surpasses loads released from adjacent areas (agriculture, forest, grassland). Since the research was performed with the use of a modeling tool (Macromodel DNS/SWAT), we were also able to successfully distinguish between two types of urban land use with high and low density of development (UHD and ULD). This differentiation has been performed using simple hydrological parameters (PERC and SURQ) provided by the model and enabled us to overcome the insufficient readability of the SWAT model results for HRU units. The obtained results underlined over 50% difference in total and unit TN and TP loads released from UHD and ULD in the individual subbasins of the studied area. Moreover, differences in temporal pattern of TN and TP loads have been presented, showing a contrasting behaviour of TN and TP over the course of the year from UHD and ULD.

Furthermore, applied climate change scenarios highlighted the future increase of the nutrient surface runoff loads from all types of land use, but most distinctly from the UHD and ULD. Since the proposed approach to distinguish between the impact of UHD and ULD could be easily adopted for any urban basin, this can offer new opportunities for the SWAT model community. This approach could be used in the context of blue-green infrastructure modeling and development of other nutrient load limitation measures.

As for the situation created by the war in Ukraine and its impact on the population migration, and consequently city development, our approach will gain even greater importance and additional applications. Diversification of urban development types allows for earlier and more precise prediction of environmental effects of single- and multi-family housing projects providing accommodation to current and future city residents. Therefore, the presented results should be taken into account by the city authorities and urban planners, who have the greatest influence on the future shape and direction of the municipality development. We have clearly shown that ULD surfaces are much better at retaining nutrients, so the greatest emphasis should be directed towards the development of blue-green infrastructure. The modeling results are also an important source of knowledge for water management planning institutions, where knowledge about the impact of land use changes in the catchment area and its effect on water quality is crucial.

### Supplementary Information

Below is the link to the electronic supplementary material.

Supplementary file1 (DOCX 1390 KB)

## Data Availability

All data supporting the findings of this study are available within the paper and its Supplementary Information.
