# Supplementary material for: Total nitrogen and phosphorus loads in surface runoff from urban land use (city of Lublin) under climate change
Source: Environ Sci Pollut Res Int. 2024 Jul 17;31(35):48135–53. doi: 10.1007/s11356-024-34365-9 (PMC11297819; doi:10.1007/s11356-024-34365-9)
Supplement: Supplementary file 1 — Supplementary file1 (DOCX 1390 KB) [file 11356_2024_34365_MOESM1_ESM.docx]

**Supporting Information for**

**Total nitrogen and phosphorus loads in surface runoff from an urban land use (city of Lublin) under climate changes**

Ewa Szalińska^a^, Elżbieta Jarosińska^b^, Paulina Orlińska-Woźniak^c^, Ewa Jakusik^c^, Wiktoria Warzecha^b^, Wioletta Ogar^b^, Paweł Wilk^c^

1. AGH University of Krakow. Address: A. Mickiewicza Av. 30, 30-059 Krakow, Poland
2. Cracow University of Technology, Address: Warszawska 24, 31-155 Cracow, Poland
3. Institute of Meteorology and Water Management - National Research Institute. Address: Podleśna 61, 01-673 Warsaw, Poland


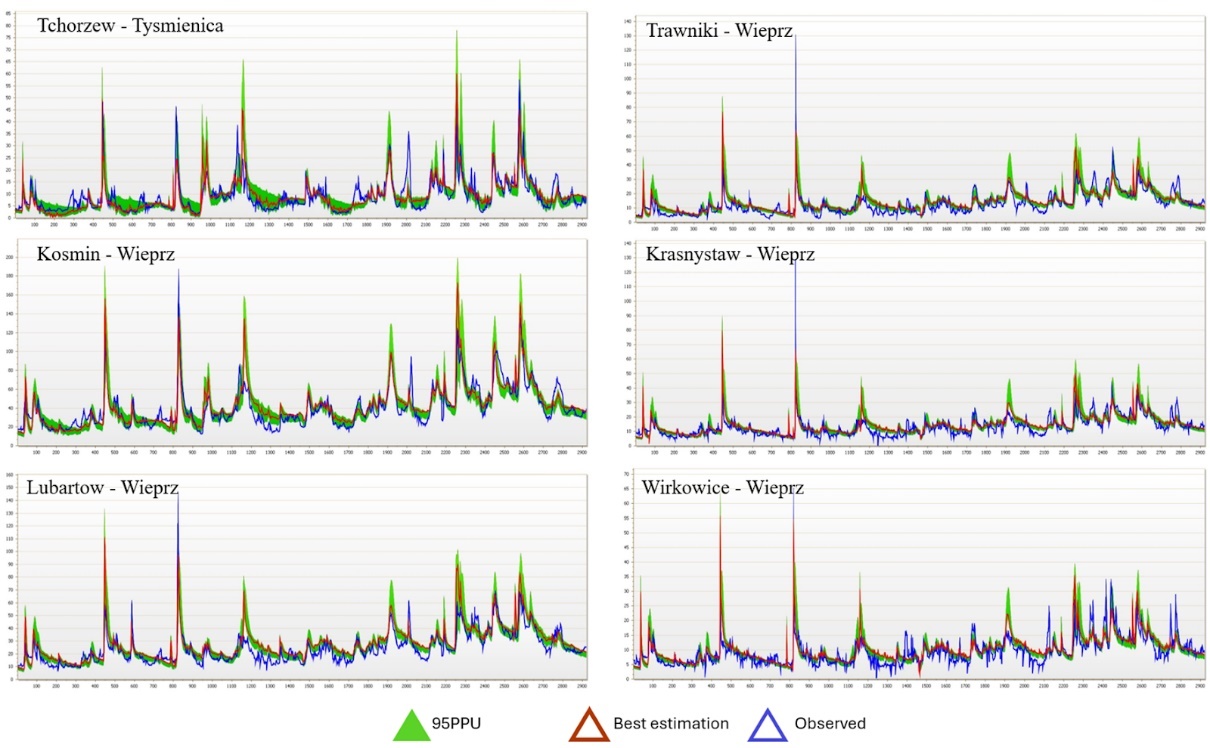


**Figure SI1.** Simulated and observed flows during the calibration period (2007-2011) for the Wieprz catchment in selected calculation profiles.


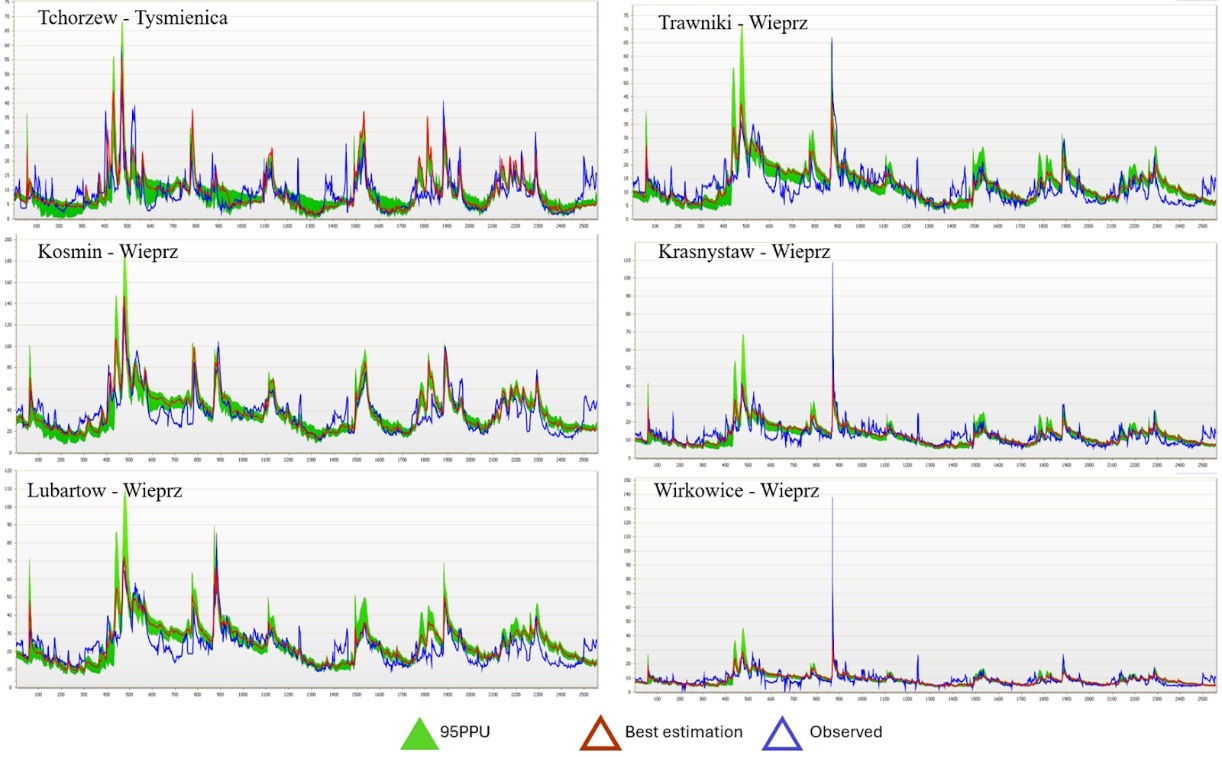


**Figure SI2.** Simulated and observed flows during the verification period (2012-2018) for the Wieprz catchment in selected calculation profiles.


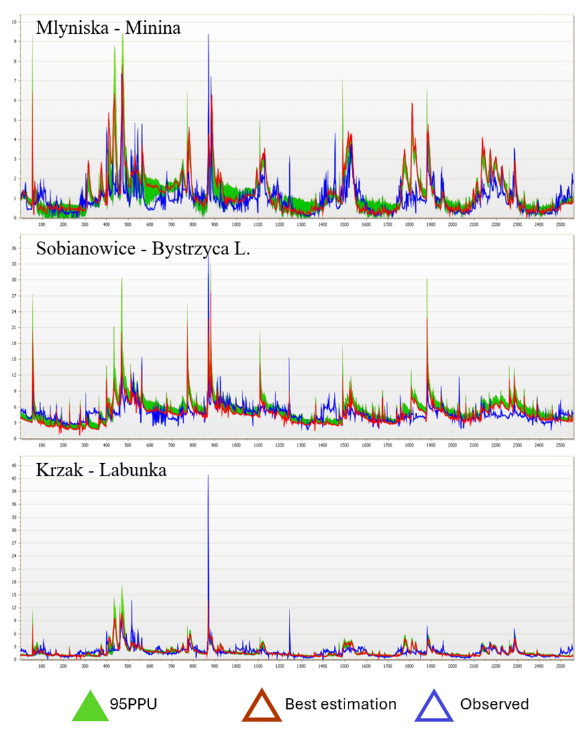


**Figure SI3.** Simulated and observed flows during the validation period (2004-2015) for the Wieprz catchment in selected calculation profiles.


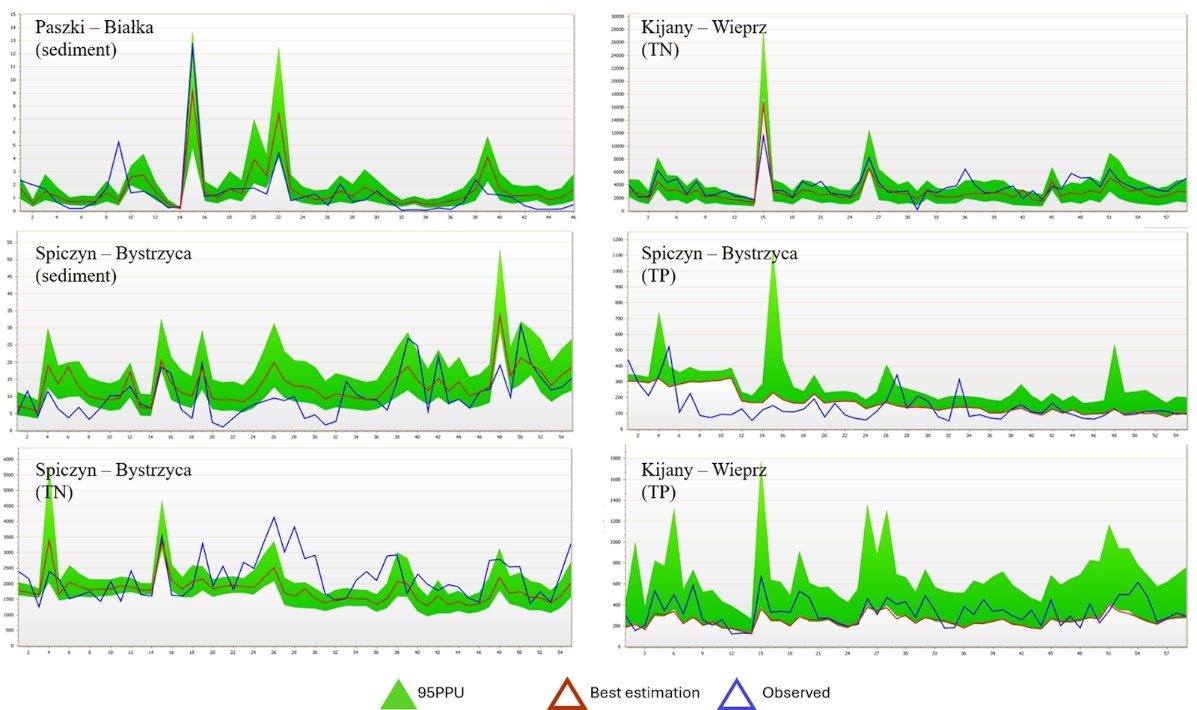


**Figure SI4.** Variability of sediment loads [t/d], total nitrogen [kg/d] and total phosphorus [kg/d] simulated and observed during the calibration period (2007-2011) for selected calculation profiles.


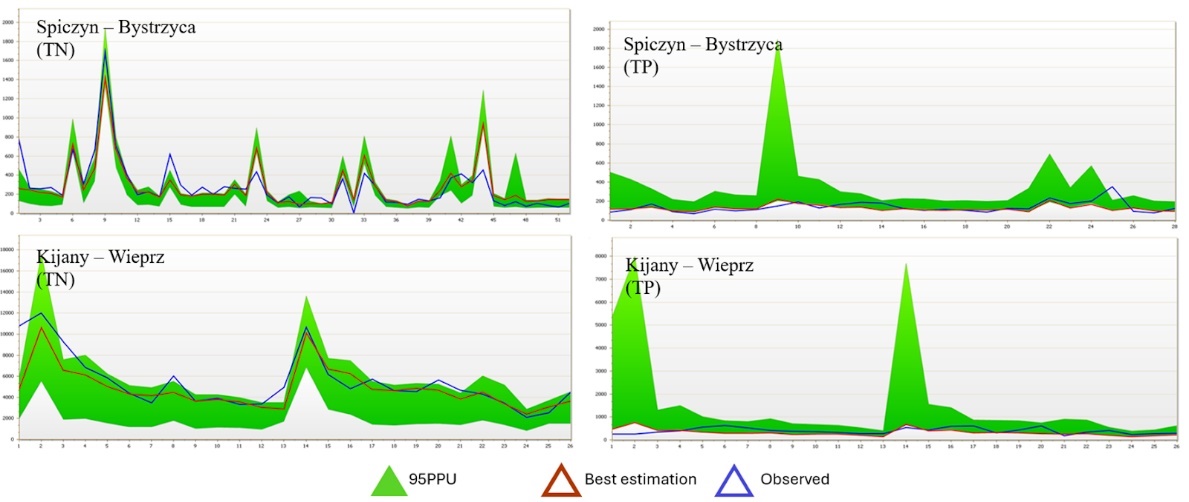


**Figure SI5.** Variability of total nitrogen [kg/d] and total phosphorus [kg/d] simulated and observed during the verification period (2012-2018) for selected calculation profiles.


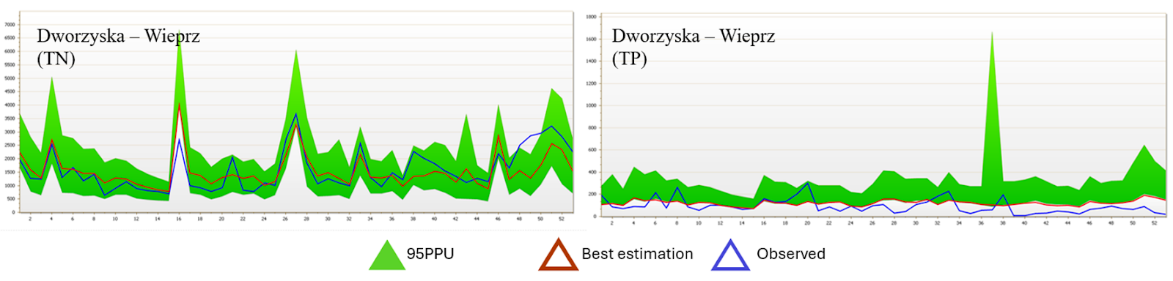


**Figure SI6.** Variability of total nitrogen [kg/d] and total phosphorus [kg/d] loads simulated and observed during the validation period (2004-2015) for selected calculation profiles.

**Table SI7.** Sensitivity analysis performed in the SUFI-2 algorithm. The smaller the value of the
p-significance level (p-value), the more sensitive the parameter. In turn, the value of the t-statistic
(t-stat) indicates the intensity and direction of change in a given parameter.

| Parameter Name | Definition | Unit | t-Stat | p-Value |
| --- | --- | --- | --- | --- |
| Flow | | | | |
| CN2.mgt | Initial SCS runoff curve number for moisture condition | - | -7.53 | 0 |
| RCHRG_DP.gw | Deep aquifer percolation fraction | - | 5.28 | 0 |
| TDRAIN.mgt | Tome to drain soil to field capacity | hours | 4.27 | 0 |
| CH_N2.rte | Manning's "n" value for the main channel | - | 3.62 | 0 |
| NDTARGR.res | Number of days to reach target storage from current reservoir storage | days | 3.1 | 0 |
| GW_DELAY.gw | Groundwater delay time | days | -2.78 | 0.01 |
| ESCO.hru | Soil evaporation compensation factor | mm | 2.61 | 0.01 |
| GDRAIN.mgt | Drain tile lag time | hours | 2.45 | 0.02 |
| GWQMN.gw | Threshold depth of water in the shallow aquifer required for return flow to occur | mm H_2_O | -2.04 | 0.04 |
| CH_K2.rte | Effective hydraulic conductivity in the main channel alluvium | mm/hours | 1.61 | 0.11 |
| SOL_AWC.sol | Available water capacity of the soil layer | mm H_2_O/mm soil | -1.37 | 0.17 |
| ALPHA_BF.gw | Baseflow alpha factor | 1/days | -1.34 | 0.18 |
| SURLAG.hru | Surface runoff lag coefficient | - | 0.51 | 0.61 |
| DEP_IMP.hru | Depth to impervious layer in soil profile | mm | 0.5 | 0.62 |
| EPCO.hru | Plant uptake compensation factor | - | 0.36 | 0.72 |
| GWQMN.gw | Threshold depth of water in the shallow aquifer required for return flow to occur | mm H_2_O | 0.25 | 0.81 |
| Nitrogen | | | | |
| ERORGN.hru | Organic N enrichment ratio for loading with sediment | - | -0.07 | 0.94 |
| NSETLR1.lwq | Nitrogen settling rate in reservoir for months | m/year | -0.6 | 0.55 |
| FIXCO.bsn | Nitrogen fixation coefficient | - | -0.86 | 0.39 |
| NSETLR2.lwq | Nitrogen settling rate in reservoir for months | m/year | -1.03 | 0.3 |
| N_UPDIS.bsn | Nitrogen uptake distribution parameter | - | 2.21 | 0.03 |
| HLIFE_NGW.gw | Half-life of nitrate in the shallow aquifer | days | -9.7 | 0 |
| NPERCO.bsn | Nitrate percolation coefficient | - | -11.68 | 0 |
| SDNCO.bsn | Denitrification threshold water content | - | -13.32 | 0 |
| SOL_ORGN.ch | Initial organic N concentration in the soil layer | mgN/kg | -21.14 | 0 |
| CMN.bsn | Rate factor for humus mineralization of active organic nutrients | - | -21.5 | 0 |
| Phosphorus | | | | |
| PSP.bsn | Phosphorus availability index | - | 0.2 | 0.84 |
| SOL_ORGP.ch | Initial organic P concentration in soil layer | mgP/kg | -0.24 | 0.81 |
| RSDCO.bsn | Residue decomposition coefficient | - | -1.05 | 0.29 |
| ERORGP.hru | Phosphorus enrichment ratio for loading with sediment | - | -1.08 | 0.28 |
| PSETLR1.lwq | Phosphorus settling rate in reservoir for months | m/year | 1.2 | 0.23 |
| SOL_LABP.ch | Initial concentration of soluble P | - | -1.23 | 0.22 |
| P_UPDIS.bsn | Phosphorus uptake distribution parameter | - | 1.77 | 0.08 |
| PSETLR2.lwq | Phosphorus settling rate in reservoir for months | m/year | 3.04 | 0 |
| RS5.swq | Organic phosphorus settling rate in the reach at 20oC | day | 5.25 | 0 |
| GWSOLP.gw | Concentration of soluble phosphorus in groundwater contribution to stream flow from subbasin | mgP/L | -14.32 | 0 |
| LAT_ORGP.gw | Organic P in the base flow | mg/l | -50.84 | 0 |

**Table SI8**. Classification of value ranges for statistical measures used during calibration, verification, and validation, based on: Moriasi et al., 2015; Patil et al., 2015; Libera et al., 2018

| Performance rating \ Parameter | Flow | TN | Sediment/TP |
| --- | --- | --- | --- |
|  | R^2^ | | |
| Very good | > 0.85 | > 0.7 | > 0.8 |
| Good | 0.75 - 0.85 | 0.6 - 0.7 | 0.65 - 0.8 |
| Satisfactory | 0.6 - 0.75 | 0.3 - 0.6 | 0.40 - 0.65 |
| Non satisfactory | < 0.5 | < 0.3 | < 0.4 |
|  | PBIAS % | | |
| Very good | <±10 | < ±15 | <±25 |
| Good | ±10 - ±15 | ±15 - ±20 | ±25 - ±40 |
| Satisfactory | ±15 - ±25 | ±20 - ±30 | ±40 - ±70 |
| Non satisfactory | ≥±25 | ≥±30 | ≥±70 |
|  | KGE | | |
| Very good | >0.75 | >0.75 | >0.75 |
| Good | 0.5 - 0.75 | 0.5 - 0.75 | 0.5 - 0.75 |
| Satisfactory | 0 - 0.5 | 0 - 0.5 | 0 - 0.5 |
| Non satisfactory | <0 | <0 | <0 |

*Moriasi, D. N., Gitau, M. W., Pai, N., Daggupati, P. (2015). Hydrologic and water quality models: Performance measures and evaluation criteria. Transactions of the ASABE, 58(6), 1763-1785. 10.13031/trans.58.10715*

*Patil, S. D., Stieglitz, M. (2015). Comparing spatial and temporal transferability of hydrological model parameters. J. Hydrol., 525, 409-417. https://doi.org/10.1016/j.jhydrol.2015.04.003*

*Libera, D. A., Sankarasubramanian, A. (2018). Multivariate bias corrections of mechanistic water quality model predictions. J. Hydrol., 564, 529-541.https://doi.org/10.1016/j.jhydrol.2018.07.043*

**Table SI9.** The Wieprz River catchment SWAT module calibration, verification and validation results for daily flow simulations.

| Calculation profile | KGE | R2 | PBIAS |
| --- | --- | --- | --- |
| calibration | | | |
| Tchorzew - Tysmienica | 0.76 | 0.63 | 0.6 |
| Kosmin - Wieprz | 0.84 | 0.77 | -1.3 |
| Lubartow - Wieprz | 0.86 | 0.77 | -6.1 |
| Trawniki - Wieprz | 0.8 | 0.68 | -7.3 |
| Krasnystaw - Wieprz | 0.8 | 0.66 | -3.2 |
| Wirkowice - Wieprz | 0.79 | 0.63 | -2.1 |
| verification | | | |
| Tchorzew - Tysmienica | 0.65 | 0.47 | 13.5 |
| Kosmin - Wieprz | 0.74 | 0.62 | 5.4 |
| Lubartow - Wieprz | 0.75 | 0.7 | 1.6 |
| Trawniki - Wieprz | 0.74 | 0.6 | 3.6 |
| Krasnystaw - Wieprz | 0.79 | 0.64 | 6.9 |
| Wirkowice - Wieprz | 0.68 | 0.52 | 5.9 |
| validation | | | |
| Mlyniska - Minina | 0.51 | 0.53 | -12.8 |
| Sobianowice - Bystrzyca L. | 0.66 | 0.43 | -3.3 |
| Krzak - Labunka | 0.66 | 0.58 | 11.1 |

**Table SI10.** The Wieprz River catchment SWAT module calibration, verification and validation results for daily TN, TP and sediment simulations.

| Calculation profile | parameter | KGE | R^2^ | PBIAS |
| --- | --- | --- | --- | --- |
| Calibration | | | | |
| Paszki - Białka | sediment [t/d] | 0.63 | 0.57 | 19.9 |
| Spiczyn - Bystrzyca |  | 0.48 | 0.38 | -26.5 |
| Spiczyn - Bystrzyca | TN [kg/d] | 0.26 | 0.17 | 18.7 |
| Kijany - Wieprz |  | 0.67 | 0.65 | 19 |
| Spiczyn - Bystrzyca | TP [kg/d] | 0.37 | 0.2 | -17.7 |
| Kijany - Wieprz |  | 0.3 | 0.43 | 24.7 |
| Verification | | | | |
| Spiczyn - Bystrzyca | TN [kg/d] | 0.84 | 0.88 | 6.7 |
| Kijany - Wieprz |  | 0.57 | 0.69 | 32.2 |
| Spiczyn - Bystrzyca | TP [kg/d] | 0.21 | 0.12 | 8.9 |
| Kijany - Wieprz |  | 0.16 | 0.03 | 18.2 |
| Validation | | | | |
| Dworzyska - Wieprz | Sediment [t/d] | 0.23 | 0.16 | -4.5 |
|  | TN [kg/d] | 0.7 | 0.55 | 1.5 |
|  | TP [kg/d] | -0.12 | 0.02 | -30.5 |

**Table SI11.** GCM-RCM model pairs applied in the current study


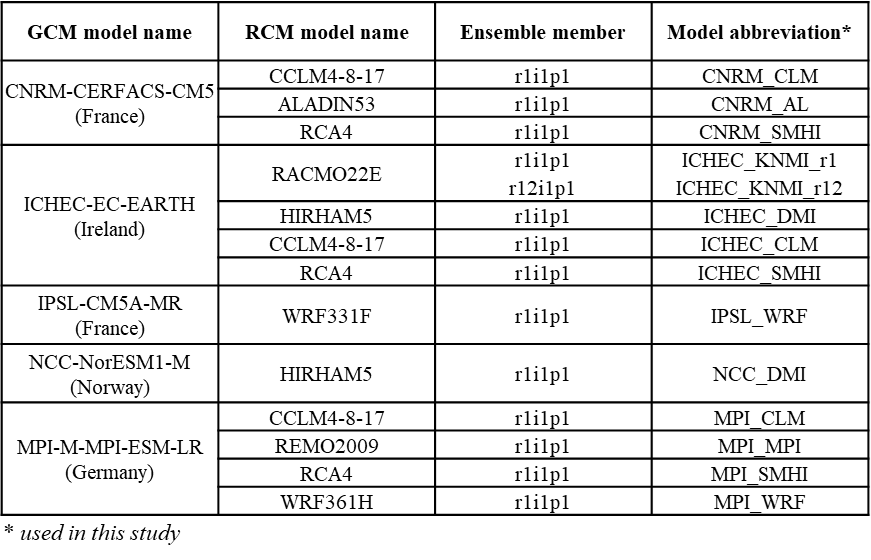


**Table SI12.** Average monthly total and unit loads of TN and TP in surface runoff from selected subbasins in the city of Lublin (kg/m and kg/ha/m) in the baseline (BS) and variant scenarios
(VS1-VS4).

| Scenario | Month | Impervious area | | | | |  | Pervious area | | | | | | | |
| --- | --- | --- | --- | --- | --- | --- | --- | --- | --- | --- | --- | --- | --- | --- | --- |
|  |  | UHD | |  | ULD | |  | Agro | |  | Forest | |  | Grassland | |
|  |  | kg/m | kg/ha/m |  | kg/m | kg/ha/m |  | kg/m | kg/ha/m |  | kg/m | kg/ha/m |  | kg/m | kg/ha/m |
|  |  | TN | | | | | | | | | | | | | |
| Baseline | 1 | 3 512 | 0.66 |  | 360 | 0.29 |  | 1 530 | 0.19 |  | 74 | 0.07 |  | 62 | 0.13 |
|  | 2 | 4 803 | 0.9 |  | 438 | 0.36 |  | 1 606 | 0.2 |  | 72 | 0.07 |  | 65 | 0.14 |
|  | 3 | 6 772 | 1.27 |  | 654 | 0.53 |  | 1 318 | 0.17 |  | 96 | 0.09 |  | 82 | 0.17 |
|  | 4 | 4 575 | 0.86 |  | 621 | 0.51 |  | 1 220 | 0.16 |  | 100 | 0.1 |  | 78 | 0.16 |
|  | 5 | 6 088 | 1.14 |  | 1 012 | 0.83 |  | 1 376 | 0.18 |  | 111 | 0.11 |  | 78 | 0.16 |
|  | 6 | 4 013 | 0.75 |  | 626 | 0.51 |  | 1 089 | 0.14 |  | 103 | 0.1 |  | 72 | 0.15 |
|  | 7 | 4 215 | 0.79 |  | 828 | 0.68 |  | 1 027 | 0.13 |  | 97 | 0.09 |  | 67 | 0.14 |
|  | 8 | 575 | 0.11 |  | 86 | 0.07 |  | 916 | 0.12 |  | 86 | 0.08 |  | 60 | 0.13 |
|  | 9 | 759 | 0.14 |  | 90 | 0.07 |  | 802 | 0.1 |  | 75 | 0.07 |  | 52 | 0.11 |
|  | 10 | 763 | 0.14 |  | 90 | 0.07 |  | 768 | 0.1 |  | 70 | 0.07 |  | 50 | 0.1 |
|  | 11 | 433 | 0.08 |  | 61 | 0.05 |  | 795 | 0.1 |  | 61 | 0.06 |  | 50 | 0.1 |
|  | 12 | 1 050 | 0.2 |  | 100 | 0.08 |  | 910 | 0.12 |  | 66 | 0.06 |  | 55 | 0.12 |
|  | av. | 3 130 | 0.59 |  | 414 | 0.34 |  | 1 113 | 0.14 |  | 84 | 0.08 |  | 64 | 0.14 |
| VS1 | 1 | 3 974 | 0.75 |  | 449 | 0.37 |  | 2 238 | 0.28 |  | 150 | 0.15 |  | 107 | 0.23 |
|  | 2 | 5 726 | 1.08 |  | 502 | 0.41 |  | 2 006 | 0.26 |  | 149 | 0.14 |  | 105 | 0.22 |
|  | 3 | 8 127 | 1.53 |  | 808 | 0.66 |  | 2 024 | 0.26 |  | 186 | 0.18 |  | 123 | 0.26 |
|  | 4 | 7 095 | 1.33 |  | 798 | 0.65 |  | 2 370 | 0.3 |  | 193 | 0.19 |  | 120 | 0.25 |
|  | 5 | 4 860 | 0.91 |  | 831 | 0.68 |  | 1 954 | 0.25 |  | 207 | 0.2 |  | 121 | 0.26 |
|  | 6 | 4 528 | 0.85 |  | 787 | 0.64 |  | 1 714 | 0.22 |  | 188 | 0.18 |  | 109 | 0.23 |
|  | 7 | 3 802 | 0.72 |  | 971 | 0.79 |  | 1 600 | 0.2 |  | 175 | 0.17 |  | 102 | 0.22 |
|  | 8 | 603 | 0.11 |  | 148 | 0.12 |  | 1 430 | 0.18 |  | 156 | 0.15 |  | 91 | 0.19 |
|  | 9 | 754 | 0.14 |  | 141 | 0.11 |  | 1 247 | 0.16 |  | 136 | 0.13 |  | 80 | 0.17 |
|  | 10 | 936 | 0.18 |  | 151 | 0.12 |  | 1 235 | 0.16 |  | 127 | 0.12 |  | 76 | 0.16 |
|  | 11 | 628 | 0.12 |  | 115 | 0.09 |  | 1 305 | 0.17 |  | 116 | 0.11 |  | 78 | 0.16 |
|  | 12 | 1 648 | 0.31 |  | 187 | 0.15 |  | 1 703 | 0.22 |  | 134 | 0.13 |  | 90 | 0.19 |
|  | av. | 3 557 | 0.67 |  | 491 | 0.4 |  | 1 736 | 0.22 |  | 160 | 0.15 |  | 100 | 0.21 |
| V2 | 1 | 4 229 | 0.8 |  | 465 | 0.38 |  | 2 201 | 0.28 |  | 156 | 0.15 |  | 109 | 0.23 |
|  | 2 | 6 371 | 1.2 |  | 569 | 0.46 |  | 2 020 | 0.26 |  | 159 | 0.15 |  | 110 | 0.23 |
|  | 3 | 7 445 | 1.4 |  | 712 | 0.58 |  | 2 158 | 0.27 |  | 202 | 0.19 |  | 131 | 0.28 |
|  | 4 | 6 840 | 1.29 |  | 735 | 0.6 |  | 2 267 | 0.29 |  | 206 | 0.2 |  | 127 | 0.27 |
|  | 5 | 4 411 | 0.83 |  | 759 | 0.62 |  | 2 008 | 0.26 |  | 211 | 0.2 |  | 124 | 0.26 |
|  | 6 | 4 776 | 0.9 |  | 1 020 | 0.83 |  | 1 740 | 0.22 |  | 190 | 0.18 |  | 111 | 0.23 |
|  | 7 | 2 790 | 0.52 |  | 684 | 0.56 |  | 1 628 | 0.21 |  | 176 | 0.17 |  | 103 | 0.22 |
|  | 8 | 569 | 0.11 |  | 152 | 0.12 |  | 1 456 | 0.19 |  | 157 | 0.15 |  | 92 | 0.19 |
|  | 9 | 749 | 0.14 |  | 145 | 0.12 |  | 1 269 | 0.16 |  | 137 | 0.13 |  | 80 | 0.17 |
|  | 10 | 1 125 | 0.21 |  | 170 | 0.14 |  | 1 351 | 0.17 |  | 130 | 0.12 |  | 78 | 0.16 |
|  | 11 | 720 | 0.14 |  | 126 | 0.1 |  | 1 413 | 0.18 |  | 124 | 0.12 |  | 84 | 0.18 |
|  | 12 | 1 561 | 0.29 |  | 194 | 0.16 |  | 1 670 | 0.21 |  | 144 | 0.14 |  | 96 | 0.2 |
|  | av. | 3 465 | 0.65 |  | 478 | 0.39 |  | 1 765 | 0.22 |  | 166 | 0.16 |  | 104 | 0.22 |
| V3 | 1 | 3 697 | 0.7 |  | 430 | 0.35 |  | 2 087 | 0.27 |  | 139 | 0.13 |  | 99 | 0.21 |
|  | 2 | 5 766 | 1.08 |  | 494 | 0.4 |  | 1 971 | 0.25 |  | 136 | 0.13 |  | 98 | 0.21 |
|  | 3 | 7 051 | 1.33 |  | 685 | 0.56 |  | 1 870 | 0.24 |  | 166 | 0.16 |  | 115 | 0.24 |
|  | 4 | 6 802 | 1.28 |  | 775 | 0.63 |  | 2 111 | 0.27 |  | 172 | 0.17 |  | 112 | 0.23 |
|  | 5 | 4 905 | 0.92 |  | 835 | 0.68 |  | 1 839 | 0.23 |  | 186 | 0.18 |  | 113 | 0.24 |
|  | 6 | 4 856 | 0.91 |  | 1 046 | 0.85 |  | 1 593 | 0.2 |  | 169 | 0.16 |  | 102 | 0.21 |
|  | 7 | 2 761 | 0.52 |  | 640 | 0.52 |  | 1 493 | 0.19 |  | 157 | 0.15 |  | 95 | 0.2 |
|  | 8 | 575 | 0.11 |  | 131 | 0.11 |  | 1 335 | 0.17 |  | 140 | 0.13 |  | 85 | 0.18 |
|  | 9 | 704 | 0.13 |  | 123 | 0.1 |  | 1 164 | 0.15 |  | 122 | 0.12 |  | 74 | 0.15 |
|  | 10 | 1 046 | 0.2 |  | 147 | 0.12 |  | 1 210 | 0.15 |  | 115 | 0.11 |  | 71 | 0.15 |
|  | 11 | 592 | 0.11 |  | 103 | 0.08 |  | 1 264 | 0.16 |  | 107 | 0.1 |  | 75 | 0.16 |
|  | 12 | 1 591 | 0.3 |  | 175 | 0.14 |  | 1 583 | 0.2 |  | 125 | 0.12 |  | 86 | 0.18 |
|  | av. | 3 362 | 0.63 |  | 465 | 0.38 |  | 1 627 | 0.21 |  | 145 | 0.14 |  | 94 | 0.2 |
| V4 | 1 | 4 257 | 0.8 |  | 466 | 0.38 |  | 2 278 | 0.29 |  | 155 | 0.15 |  | 109 | 0.23 |
|  | 2 | 6 120 | 1.15 |  | 646 | 0.53 |  | 2 127 | 0.27 |  | 159 | 0.15 |  | 110 | 0.23 |
|  | 3 | 7 214 | 1.36 |  | 686 | 0.56 |  | 2 108 | 0.27 |  | 196 | 0.19 |  | 129 | 0.27 |
|  | 4 | 6 600 | 1.24 |  | 736 | 0.6 |  | 2 341 | 0.3 |  | 200 | 0.19 |  | 125 | 0.26 |
|  | 5 | 4 676 | 0.88 |  | 814 | 0.66 |  | 2 123 | 0.27 |  | 213 | 0.21 |  | 125 | 0.26 |
|  | 6 | 5 299 | 1 |  | 1 141 | 0.93 |  | 1 812 | 0.23 |  | 194 | 0.19 |  | 113 | 0.24 |
|  | 7 | 1 602 | 0.3 |  | 324 | 0.26 |  | 1 694 | 0.22 |  | 180 | 0.17 |  | 106 | 0.22 |
|  | 8 | 675 | 0.13 |  | 155 | 0.13 |  | 1 518 | 0.19 |  | 161 | 0.16 |  | 94 | 0.2 |
|  | 9 | 731 | 0.14 |  | 140 | 0.11 |  | 1 323 | 0.17 |  | 140 | 0.14 |  | 82 | 0.17 |
|  | 10 | 1 048 | 0.2 |  | 161 | 0.13 |  | 1 367 | 0.17 |  | 132 | 0.13 |  | 79 | 0.17 |
|  | 11 | 697 | 0.13 |  | 120 | 0.1 |  | 1 430 | 0.18 |  | 123 | 0.12 |  | 84 | 0.18 |
|  | 12 | 1 422 | 0.27 |  | 177 | 0.14 |  | 1 649 | 0.21 |  | 140 | 0.13 |  | 94 | 0.2 |
|  | av. | 3 362 | 0.63 |  | 464 | 0.38 |  | 1 814 | 0.23 |  | 166 | 0.16 |  | 104 | 0.22 |
|  |  | TP | | | | | | | | | | | | | |
| Baseline | 1 | 115 | 0.022 |  | 9 | 0.008 |  | 110 | 0.014 |  | 3 | 0.003 |  | 2 | 0.005 |
|  | 2 | 202 | 0.038 |  | 18 | 0.015 |  | 118 | 0.015 |  | 3 | 0.003 |  | 2 | 0.005 |
|  | 3 | 245 | 0.046 |  | 19 | 0.015 |  | 52 | 0.007 |  | 3 | 0.003 |  | 3 | 0.006 |
|  | 4 | 217 | 0.041 |  | 21 | 0.017 |  | 45 | 0.006 |  | 4 | 0.003 |  | 3 | 0.006 |
|  | 5 | 716 | 0.135 |  | 99 | 0.081 |  | 69 | 0.009 |  | 5 | 0.004 |  | 3 | 0.007 |
|  | 6 | 264 | 0.05 |  | 29 | 0.024 |  | 39 | 0.005 |  | 4 | 0.004 |  | 3 | 0.005 |
|  | 7 | 328 | 0.062 |  | 31 | 0.025 |  | 37 | 0.005 |  | 3 | 0.003 |  | 2 | 0.005 |
|  | 8 | 144 | 0.027 |  | 13 | 0.011 |  | 32 | 0.004 |  | 3 | 0.003 |  | 2 | 0.004 |
|  | 9 | 208 | 0.039 |  | 19 | 0.016 |  | 29 | 0.004 |  | 3 | 0.003 |  | 2 | 0.004 |
|  | 10 | 203 | 0.038 |  | 19 | 0.015 |  | 28 | 0.004 |  | 2 | 0.002 |  | 2 | 0.004 |
|  | 11 | 97 | 0.018 |  | 8 | 0.006 |  | 29 | 0.004 |  | 2 | 0.002 |  | 2 | 0.004 |
|  | 12 | 177 | 0.033 |  | 13 | 0.011 |  | 36 | 0.005 |  | 2 | 0.002 |  | 2 | 0.004 |
|  | av. | 243 | 0.046 |  | 25 | 0.02 |  | 52 | 0.007 |  | 3 | 0.003 |  | 2 | 0.005 |
| VS1 | 1 | 131 | 0.025 |  | 13 | 0.01 |  | 135 | 0.017 |  | 5 | 0.005 |  | 4 | 0.009 |
|  | 2 | 268 | 0.05 |  | 23 | 0.019 |  | 110 | 0.014 |  | 5 | 0.005 |  | 4 | 0.008 |
|  | 3 | 271 | 0.051 |  | 23 | 0.019 |  | 81 | 0.01 |  | 7 | 0.006 |  | 4 | 0.009 |
|  | 4 | 596 | 0.112 |  | 58 | 0.048 |  | 138 | 0.018 |  | 7 | 0.007 |  | 4 | 0.009 |
|  | 5 | 493 | 0.093 |  | 68 | 0.055 |  | 80 | 0.01 |  | 8 | 0.007 |  | 4 | 0.009 |
|  | 6 | 346 | 0.065 |  | 42 | 0.034 |  | 64 | 0.008 |  | 7 | 0.007 |  | 4 | 0.008 |
|  | 7 | 299 | 0.056 |  | 30 | 0.024 |  | 58 | 0.007 |  | 6 | 0.006 |  | 4 | 0.008 |
|  | 8 | 142 | 0.027 |  | 15 | 0.012 |  | 51 | 0.007 |  | 6 | 0.006 |  | 3 | 0.007 |
|  | 9 | 196 | 0.037 |  | 19 | 0.016 |  | 45 | 0.006 |  | 5 | 0.005 |  | 3 | 0.006 |
|  | 10 | 261 | 0.049 |  | 25 | 0.021 |  | 49 | 0.006 |  | 4 | 0.004 |  | 3 | 0.006 |
|  | 11 | 150 | 0.028 |  | 13 | 0.01 |  | 53 | 0.007 |  | 4 | 0.004 |  | 3 | 0.006 |
|  | 12 | 352 | 0.066 |  | 31 | 0.026 |  | 91 | 0.012 |  | 5 | 0.005 |  | 3 | 0.007 |
|  | av. | 292 | 0.055 |  | 30 | 0.024 |  | 80 | 0.01 |  | 6 | 0.006 |  | 4 | 0.008 |
| V2 | 1 | 131 | 0.025 |  | 12 | 0.01 |  | 120 | 0.015 |  | 6 | 0.005 |  | 4 | 0.008 |
|  | 2 | 324 | 0.061 |  | 27 | 0.022 |  | 100 | 0.013 |  | 6 | 0.006 |  | 4 | 0.008 |
|  | 3 | 316 | 0.059 |  | 27 | 0.022 |  | 85 | 0.011 |  | 7 | 0.007 |  | 5 | 0.01 |
|  | 4 | 505 | 0.095 |  | 47 | 0.038 |  | 109 | 0.014 |  | 7 | 0.007 |  | 5 | 0.01 |
|  | 5 | 440 | 0.083 |  | 59 | 0.048 |  | 79 | 0.01 |  | 8 | 0.008 |  | 4 | 0.009 |
|  | 6 | 253 | 0.048 |  | 30 | 0.025 |  | 63 | 0.008 |  | 7 | 0.007 |  | 4 | 0.008 |
|  | 7 | 297 | 0.056 |  | 29 | 0.024 |  | 59 | 0.007 |  | 6 | 0.006 |  | 4 | 0.008 |
|  | 8 | 130 | 0.024 |  | 14 | 0.012 |  | 52 | 0.007 |  | 6 | 0.005 |  | 3 | 0.007 |
|  | 9 | 192 | 0.036 |  | 19 | 0.016 |  | 45 | 0.006 |  | 5 | 0.005 |  | 3 | 0.006 |
|  | 10 | 335 | 0.063 |  | 32 | 0.026 |  | 62 | 0.008 |  | 5 | 0.005 |  | 3 | 0.006 |
|  | 11 | 174 | 0.033 |  | 14 | 0.012 |  | 57 | 0.007 |  | 5 | 0.004 |  | 3 | 0.006 |
|  | 12 | 337 | 0.063 |  | 29 | 0.024 |  | 74 | 0.009 |  | 5 | 0.005 |  | 3 | 0.007 |
|  | av. | 286 | 0.054 |  | 28 | 0.023 |  | 75 | 0.01 |  | 6 | 0.006 |  | 4 | 0.008 |
| V3 | 1 | 119 | 0.022 |  | 12 | 0.01 |  | 125 | 0.016 |  | 5 | 0.005 |  | 4 | 0.008 |
|  | 2 | 254 | 0.048 |  | 21 | 0.017 |  | 112 | 0.014 |  | 5 | 0.005 |  | 4 | 0.008 |
|  | 3 | 231 | 0.043 |  | 19 | 0.016 |  | 72 | 0.009 |  | 6 | 0.006 |  | 4 | 0.009 |
|  | 4 | 562 | 0.106 |  | 55 | 0.045 |  | 114 | 0.014 |  | 6 | 0.006 |  | 4 | 0.008 |
|  | 5 | 497 | 0.094 |  | 70 | 0.057 |  | 76 | 0.01 |  | 7 | 0.007 |  | 4 | 0.009 |
|  | 6 | 290 | 0.054 |  | 34 | 0.028 |  | 59 | 0.007 |  | 6 | 0.006 |  | 4 | 0.008 |
|  | 7 | 301 | 0.057 |  | 29 | 0.024 |  | 53 | 0.007 |  | 6 | 0.005 |  | 3 | 0.007 |
|  | 8 | 138 | 0.026 |  | 14 | 0.012 |  | 48 | 0.006 |  | 5 | 0.005 |  | 3 | 0.006 |
|  | 9 | 181 | 0.034 |  | 18 | 0.014 |  | 42 | 0.005 |  | 4 | 0.004 |  | 3 | 0.006 |
|  | 10 | 310 | 0.058 |  | 30 | 0.024 |  | 52 | 0.007 |  | 4 | 0.004 |  | 3 | 0.005 |
|  | 11 | 140 | 0.026 |  | 12 | 0.01 |  | 49 | 0.006 |  | 4 | 0.004 |  | 3 | 0.006 |
|  | 12 | 335 | 0.063 |  | 30 | 0.024 |  | 78 | 0.01 |  | 5 | 0.004 |  | 3 | 0.006 |
|  | av. | 280 | 0.053 |  | 29 | 0.023 |  | 73 | 0.009 |  | 5 | 0.005 |  | 3 | 0.007 |
| V4 | 1 | 141 | 0.027 |  | 13 | 0.011 |  | 127 | 0.016 |  | 6 | 0.005 |  | 4 | 0.008 |
|  | 2 | 304 | 0.057 |  | 27 | 0.022 |  | 111 | 0.014 |  | 6 | 0.006 |  | 4 | 0.008 |
|  | 3 | 297 | 0.056 |  | 26 | 0.021 |  | 78 | 0.01 |  | 7 | 0.007 |  | 5 | 0.01 |
|  | 4 | 526 | 0.099 |  | 53 | 0.043 |  | 121 | 0.015 |  | 7 | 0.007 |  | 4 | 0.009 |
|  | 5 | 508 | 0.096 |  | 77 | 0.063 |  | 91 | 0.012 |  | 8 | 0.008 |  | 5 | 0.01 |
|  | 6 | 272 | 0.051 |  | 32 | 0.026 |  | 66 | 0.008 |  | 7 | 0.007 |  | 4 | 0.009 |
|  | 7 | 298 | 0.056 |  | 29 | 0.023 |  | 61 | 0.008 |  | 6 | 0.006 |  | 4 | 0.008 |
|  | 8 | 163 | 0.031 |  | 17 | 0.014 |  | 55 | 0.007 |  | 6 | 0.006 |  | 3 | 0.007 |
|  | 9 | 185 | 0.035 |  | 19 | 0.015 |  | 47 | 0.006 |  | 5 | 0.005 |  | 3 | 0.006 |
|  | 10 | 305 | 0.057 |  | 30 | 0.024 |  | 58 | 0.007 |  | 5 | 0.005 |  | 3 | 0.006 |
|  | 11 | 161 | 0.03 |  | 13 | 0.011 |  | 56 | 0.007 |  | 5 | 0.004 |  | 3 | 0.006 |
|  | 12 | 310 | 0.058 |  | 28 | 0.023 |  | 70 | 0.009 |  | 5 | 0.005 |  | 3 | 0.007 |
|  | av. | 289 | 0.054 |  | 30 | 0.025 |  | 79 | 0.01 |  | 6 | 0.006 |  | 4 | 0.008 |
